# Controlled Epitaxial Growth of Perovskite Single-Crystal Heterojunction Arrays for Self-Powered Imaging

**DOI:** 10.1007/s40820-026-02224-6

**Published:** 2026-05-29

**Authors:** Hui Lu, Yang Yu, Wenqiang Wu, Zeping He, Kaiyu Hu, Wenqiang Yang, Xun Han, Caofeng Pan

**Affiliations:** 1 CAS Center for Excellence in Nanoscience, Beijing Key Laboratory of Micro-Nano Energy and Sensor, Beijing Institute of Nanoenergy and Nanosystems, Chinese Academy of Sciences, Beijing, 101400 People’s Republic of China; 2 Beijing Key Laboratory for Atomic Manufacturing Equipment and Intelligent Sensing, Institute of Atomic Manufacturing, Beihang University, Beijing, 100191 People’s Republic of China; 3 School of Nanoscience and Technology, University of Chinese Academy of Sciences, Beijing, 100049 People’s Republic of China; 4 Department of Applied Physics, The Hong Kong Polytechnic University, Hong Kong, 999077 People’s Republic of China

**Keywords:** Perovskite single crystals, Heterojunction arrays, Selective epitaxial growth, Orientation control, Self-powered imaging

## Abstract

**Highlights:**

- A versatile selective epitaxial growth strategy was developed for fabricating perovskite single-crystal heterojunction arrays, enabling precise control over pixel size, arrangement angle, and crystal orientation.
- The self-powered photodetector arrays based on the single-crystal heterojunction exhibited high sensitivity with a weak-light detection limit of 9 nW cm^−2^, long-term operational stability, and clear imaging capability under zero bias.

**Abstract:**

Perovskite single-crystal heterojunction arrays exhibit significant application potential in advanced optoelectronics, however, achieving comprehensive control over crystallographic and spatial properties of the array remains challenging. Here, we report a selective epitaxial growth strategy for fabricating single-crystal MAPbCl_3_/MAPbBr_3_ and MAPbBr_3_/MAPbI_3_ heterojunction arrays. This method employs patterned polymer templates to define the pixel dimension and arrangement, while the underlying single-crystal substrate guides the crystal orientation of the heterojunction array, enabling precise control over the pixel size, pixel arrangement angle and crystal plane. The self-powered photodetector arrays were fabricated based on these heterojunctions, showing a specific detectivity of 6.0 × 10^11^ Jones, a weak-light detection limit of 9 nW cm^−2^ and long-term operation stability under zero bias. Furthermore, the light pattern with different illumination intensities could be clearly imaged by the device array in the self-powered mode. This work establishes a robust method of fabricating the single-crystal heterojunction arrays for advanced optoelectronic applications.

**Figure:**
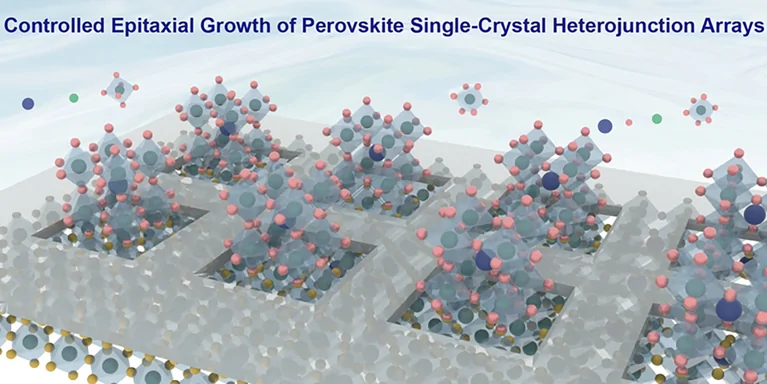

**Supplementary Information:**

The online version contains supplementary material available at 10.1007/s40820-026-02224-6.

## Introduction

Metal halide perovskites have emerged as highly promising materials for optoelectronic applications, including solar cells [1–6], light-emitting diodes [7–12], photodetectors [13–21], lasers [22–27], and neuromorphic devices [28–33]. The perovskite single crystals exhibit low trap-state densities, high carrier mobilities, and enhanced intrinsic stability compared to the polycrystalline perovskite films [34–37]. These properties make them an ideal platform for the high-performance optoelectronic devices. For the advanced applications, such as the high-resolution imaging, display and integrated optical systems, the fabrication of the perovskite single crystal into ordered array is highly required [13, 16, 20, 38–40]. Furthermore, creating single-crystal heterojunctions by interfacing two different perovskite materials is a critical step for endowing new functions and enhancing device performances [41–44]. The engineered band alignment at the interface can create the built-in electric field, which could facilitate efficient charge separation and enable self-powered optoelectronic devices [45–47].

Numerous progress has been made for the fabrication of the heterojunctions on bulk crystals or in layered films, such as utilizing the type-I band arrangement formed by quasi-2D perovskite and 3D perovskite to improve the luminous efficiency of LEDs and using partial ion exchange to prepare transverse heterojunctions with different halogen components in the type-II band alignment to improve the response capability of photodetectors [48–53]. However, the fabrication of large-scale heterojunction arrays of perovskite single crystals has not been explored. This is due to the gap between the bulk fabrication and array integration of the heterojunction, which requires miniaturizing and spatially confining the liquid-phase heterojunction formation process to construct discrete and uniform small pixels with desired pattern. Moreover, there is lack of crystallographic control methods across the heterojunction array, which often results in randomly oriented crystals, leading to severe pixel-to-pixel variations. Therefore, a method that ensures every single heterojunction pixel maintains the same orientation is urgently needed.

Here, we introduced a selective epitaxial growth approach for the perovskite single-crystal heterojunction arrays. This approach utilizes a patterned polymer template to precisely define the patterns for heterojunction formation, while arranging the crystal orientation of the entire heterojunction array to be coherently locked to that of the epitaxial substrate. Based on this strategy, the MAPbCl_3_/MAPbBr_3_ and MAPbBr_3_/MAPbI_3_ single-crystal heterojunction arrays were fabricated with precise control over pixel size, pixel angle, and crystal orientation. Vertically aligned perovskite single-crystal heterojunction photodetector arrays were further demonstrated, showing a weak-light detection limit of 9 nW cm^−2^ and a high specific detectivity of 6.0 × 10^11^ Jones at 0 V bias. Due to the uniform pixel photoresponse, the device exhibited clear pattern imaging capabilities across varying light intensities in the self-powered mode. This work provides a simple method for heterogeneous integration of perovskite single crystal arrays toward the high-performance optoelectronic devices.

## Experimental Section

### Materials

N,N-Dimethylformamide (DMF) (99%), dimethyl sulfoxide (DMSO) (99%), γ-butyrolactone (GBL) (99.9%), dipropyl sulfoxide imide (DPSI) (99.9%), methylammonium iodide (99.99%), lead iodide (99.9%), lead bromide (99.9%), lead chloride (99.9%), acetone (99%), and anhydrous ethanol (99.9%) were purchased from Aladdin Scientific. All commercial products were used inside a nitrogen-filled glove box.

### Fabrication of the Self-powered Photodetector Arrays

The fabrication of self-powered perovskite photodetector arrays based on MAPbCl_3_/MAPbBr_3_ and MAPbBr_3_/MAPbI_3_ heterojunctions was initiated by synthesizing MAPbCl_3_ and MAPbBr_3_ single crystals through a spatial confinement growth method and temperature-programmed crystallization from their respective precursor solutions dissolved in DMF/DMSO and DMF. Subsequently, a patterned Parylene-C template with selectively exposed regions was fabricated on the substrate via sequential coating, photolithography, and dry etching processes. Heteroepitaxial growth was then performed within these openings: the MAPbCl_3_/MAPbBr_3_ heterojunction was fabricated by growing MAPbBr_3_ crystals via solution epitaxy from a MAPbBr_3_-DMF precursor, whereas the MAPbBr_3_/MAPbI_3_ heterojunction was formed by epitaxially growing MAPbI_3_ crystals from a MAPbI_3_-GBL precursor. Finally, the device integration was completed by depositing electrode arrays through thermal evaporation with a shadow mask, enabling the realization of self-powered photodetector arrays.

## Results and Discussion

### Selective Epitaxial Growth of Perovskite Single-Crystal Heterojunction Arrays

**Fig. 1:**
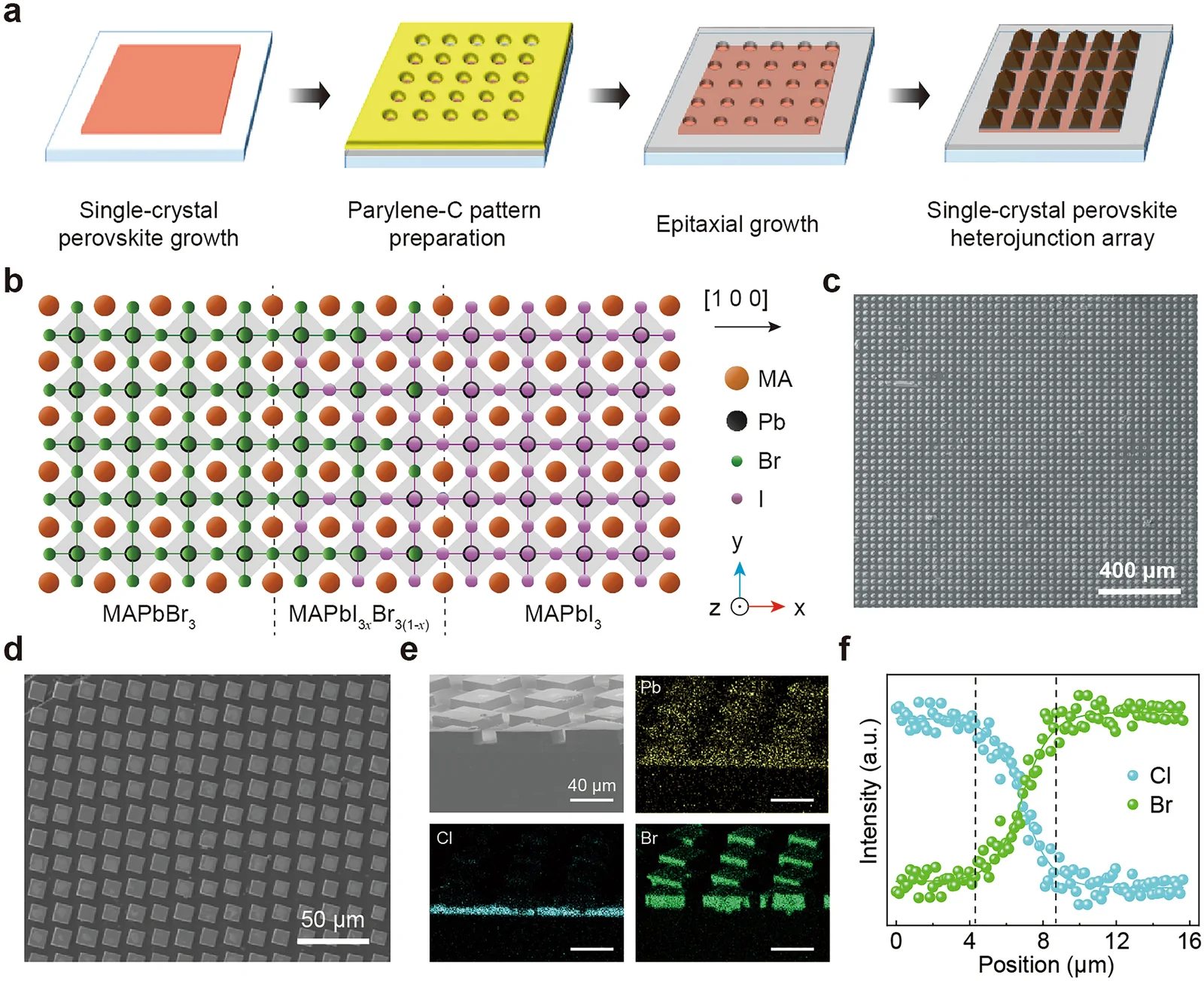
Schematic illustration of selective epitaxial growth of perovskite single-crystal heterojunction arrays. **a** Schematic of the fabrication process for the patterned perovskite single-crystal heterojunction arrays. **b** Mechanism of epitaxial growth of perovskite single-crystal heterojunction arrays. **c** Schematic diagram of the ideal lattice-matched epitaxy between MAPbBr_3_/MAPbI_3_ along the [100] crystallographic direction. **d** SEM image of a large-scale MAPbBr_3_/MAPbI_3_ single-crystal heterojunction array (44 × 44 array). **e** SEM image of MAPbCl_3_/MAPbBr_3_ single-crystal heterojunction array and EDS map of the MAPbCl_3_/MAPbBr_3_ single-crystal heterojunction array. **f** Distribution of halogen elements across the MAPbCl_3_/MAPbBr_3_ single-crystal heterojunction

The fabrication process for the patterned perovskite single-crystal heterojunction arrays is schematically illustrated in Fig. 1a. The process begins with the growth of single-crystal perovskite films on indium tin oxide (ITO) glass via a spatial confinement method, which serve as the substrates for subsequent epitaxial growth. Then, a thin Parylene-C film is deposited and patterned on the single-crystal perovskite substrate, creating openings with defined positions and sizes for the crystal epitaxial growth. Finally, the ordered single-crystal heterojunction array is formed in the openings of the Parylene-C film through the selective epitaxial growth. A more detailed fabrication process can be found in Fig. S1 and the Experimental section. Two different heterojunction arrays were fabricated, including the methylammonium lead iodide on a bromide substrate (MAPbBr_3_/MAPbI_3_) and methylammonium lead bromide on a chloride substrate (MAPbCl_3_/MAPbBr_3_). The selective epitaxial growth on the perovskite substrate involves the three stages which are different from those for the growth of a continuous perovskite film, typically involving precursor supersaturation, nucleation, and crystal growth (Fig. S2). For the selective epitaxial growth of the array, upon introduction of the precursor solution, halide ion exchange occurs in the surface region of the substrate exposed within the Parylene-C film openings. This creates a graded transition layer, such as the MAPbI_3*x*_Br_3(1-*x*)_ film on the MAPbBr_3_ surface, which also facilitates lattice-matched nucleation and homoepitaxial growth of the target MAPbBr_3_ arrays (Fig. 1b). The epitaxial growth of MAPbI_3_ on MAPbBr_3_ is achieved along the [1 0 0] crystal direction. Notably, nucleation occurs uniformly for those continuous thin films, but preferentially along crystal edges for ordered MAPbI_3_ arrays (Fig. S3). This selective epitaxial growth enables the fabrication of large-scale and high-density single-crystal arrays. Figure 1c shows a scanning electron microscopy (SEM) image of a large-scale MAPbI_3_/MAPbBr_3_ single-crystal heterostructure array, which exhibits a highly ordered and uniform arrangement array, comprising 44 × 44 individual heterojunctions within a 1.5 × 1.5 mm^2^ area. Similarly, the MAPbCl_3_/MAPbBr_3_ single-crystal heterojunction arrays were fabricated, demonstrating the uniform orientation and size as well as the sharp edges of the individual pixel (Fig. 1d). To investigate the structure and elemental distribution of the heterojunction arrays, the energy-dispersive spectroscopy (EDS) elemental mapping was performed. As shown in Fig. 1e, Cl is mainly distributed in the bottom substrate layer and Br is confined to the top epitaxial layer, while Pb demonstrates a homogeneous distribution throughout the entire structure. A line scan across the heterojunction confirms the element distribution (Fig. 1f). The concentrations of Cl and Br change gradually across the heterojunction, forming a graded transition region with a width of approximately 4 µm. This wide and graded junction confirms the initial ion-exchange process before the bulk crystal growth in Fig. S2. Similar compositional grading was also observed in the MAPbBr_3_/MAPbI_3_ heterojunction (Fig. S4).

### Epitaxial Growth Dynamics of Perovskite Single-Crystal Heterojunctions

**Fig. 2:**
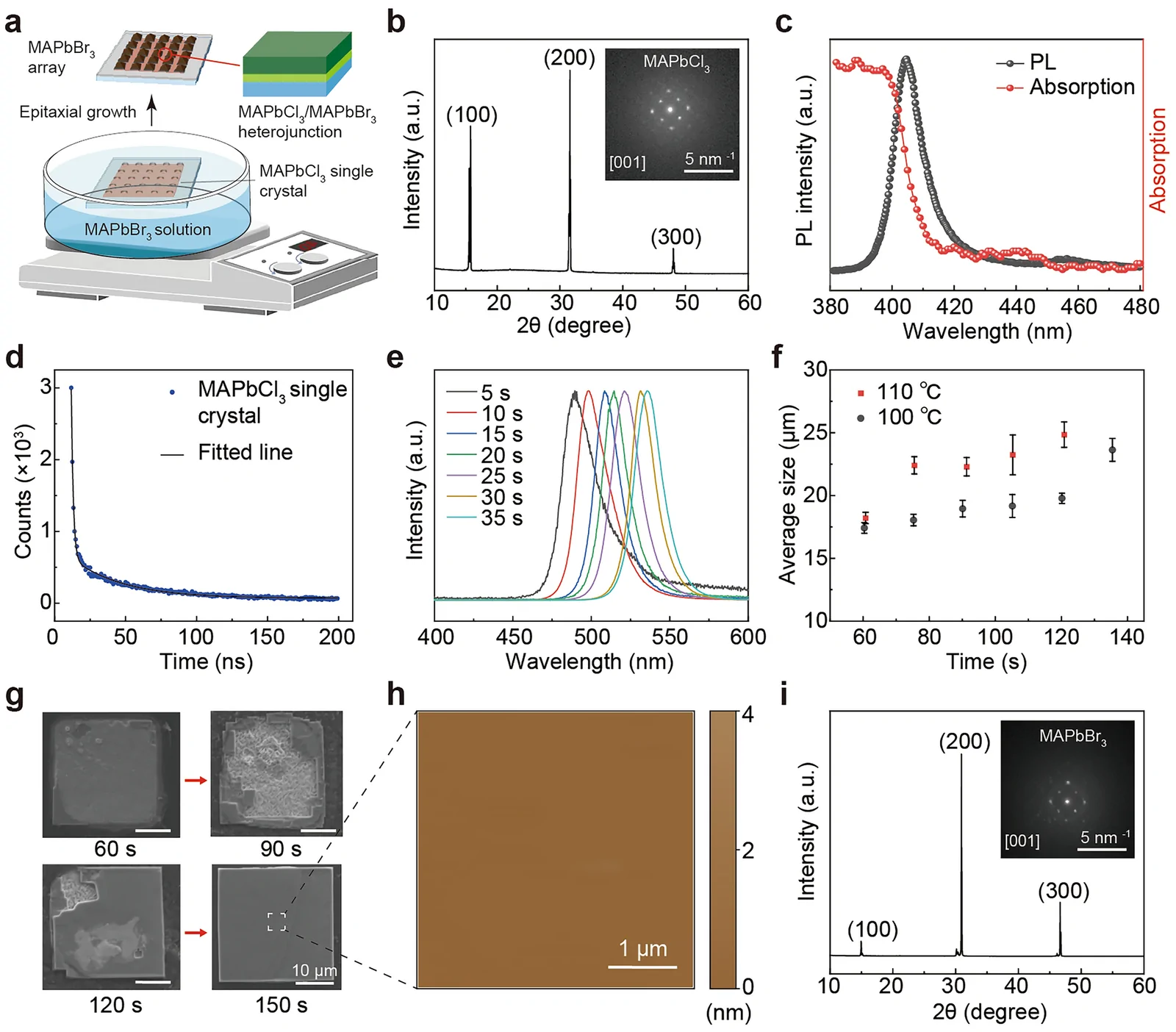
Growth dynamics of MAPbCl_3_/MAPbBr_3_ perovskite single-crystal heterojunction arrays. **a** Schematic of the experimental setup for MAPbCl_3_/MAPbBr_3_ perovskite single-crystal heterojunction arrays. **b** XRD pattern of MAPbCl_3_ single crystal substrate. Inset: SAED pattern. **c** PL and absorption spectra of MAPbCl_3_ single crystal substrate. **d** TRPL decay curve of MAPbCl_3_ single crystal substrate. **e** PL spectra of MAPbCl_3_/MAPbBr_3_ perovskite single-crystal heterojunction at different growth durations. **f** Average array size versus growth temperature and duration. **g** SEM images of epitaxial MAPbBr_3_ perovskite grown at different durations. **h** AFM image of an epitaxial MAPbBr_3_ single crystal pixel. **i** XRD pattern of the epitaxial MAPbBr_3_ single crystal array. Inset: SAED pattern

To elucidate the epitaxial growth dynamics, the epitaxial growth of the MAPbBr_3_ on the MAPbCl_3_ substrate was investigated. MAPbCl_3_/MAPbBr_3_ perovskite heterojunctions were prepared via a saturation concentration liquid-phase epitaxial growth in the heated MAPbBr_3_ precursor solution (Fig. 2a). Before the epitaxial process, MAPbCl_3_ crystals, utilized as the substrate, are obtained using a spatial confinement method liquid-phase diffusion crystallization process. The X-ray diffraction (XRD) pattern of the MAPbCl_3_ substrate displays a series of strong (100), (200), and (300) peaks, indicating the cubic phase with a preferred orientation along the < 100 > direction (Figure. 2b). The inset SAED (selected area electron diffraction) pattern further confirms the single-crystal properties of the substrate. The emission and absorption properties of MAPbCl_3_ substrate were also investigated (Fig. 2c). The photoluminescence (PL) peak is centered at 405 nm and the absorption band edge is at 3.02 eV. The charge-carrier dynamics of the MAPbCl_3_ single crystal was analyzed by time-resolved PL (TRPL) spectroscopy. The TRPL decay curve is fitted with a double-exponential fitting model with a fast decay time and a slow decay time of 1.43 and 36.53 ns, respectively (Fig. 2d). The fast decay process is caused by the bimolecular recombination of photogenerated free carriers, whereas the slow decay process is attributed mainly to trap-assisted recombination. These properties confirm the high-quality of the MAPbCl_3_ single crystal substrate.

The evolution of the heterojunction formation was monitored by tracking the PL emission of the sample at different growth intervals. As shown in Fig. 2e, a systematic redshift of the PL peak is observed as the growth time increases from 5 to 35 s. This redshift is consistent with a halide exchange process, leading to the formation of mixed halide perovskites with progressively increasing Br content. To avoid the formation of stray crystals and obtain high-quality heterojunctions, the epitaxial growth rate was optimized by maintaining the crystallization temperature within a narrow range of 100 to 110 °C. The MAPbBr_3_ arrays can be observed after about 1 min of epitaxial growth. The average sizes of the individual pixels with the growth time under different growth temperatures are summarized in Fig. 2f. The pixel size increases with growth time at both temperatures, eventually reaching and slightly exceeding the Parylene-C opening size of 20 µm. Furthermore, a higher temperature can accelerate the growth rate, leading to a larger pixel size compared to the growth at a lower temperature over the same period. Similar growth processes were also observed for the MAPbBr_3_/MAPbI_3_ heterojunction arrays (Fig. S5). Figure 2g demonstrates the morphological evolution of an individual MAPbBr_3_ perovskite crystal pixel in MAPbCl_3_/MAPbBr_3_ heterojunction arrays under 110 °C conditions. Initially, the small crystallites nucleate within the opening and after 150 s, they fully merge into a square pixel with sharp edges and a flat surface. An atomic force microscopy (AFM) image of a MAPbBr_3_ perovskite pixel confirms the smooth surface morphology, with a roughness of 2.082 nm in a 20 × 20 µm^2^ area (Fig. 2h). The crystal structure of the epitaxial MAPbBr_3_ perovskite array was characterized by XRD analysis. As shown in Fig. 2i, the XRD pattern of the MAPbBr_3_ perovskite array also demonstrates the strong diffraction peaks of (100), (200), and (300) and the SAED patterns along the [001] zone axis further confirms the high crystallinity and cubic phase of the MAPbBr_3_ perovskite array. During the transition from MAPbCl_3_ to MAPbBr_3_, the primary change is the expansion of the lattice parameter due to the larger ionic radius of Br^−^ compared to Cl^−^. This results in a shift of the XRD diffraction peaks toward lower 2θ values, consistent with Bragg’s law (Fig. 2i). The SAED pattern of the epitaxial MAPbBr_3_ array along the [001] direction is similar to that of the MAPbCl_3_ substrate, indicating that the crystal symmetry is preserved during the halogen substitution.

### Regulation of Geometric and Crystallographic Properties of Epitaxial Perovskite Single-Crystal Arrays

**Fig. 3:**
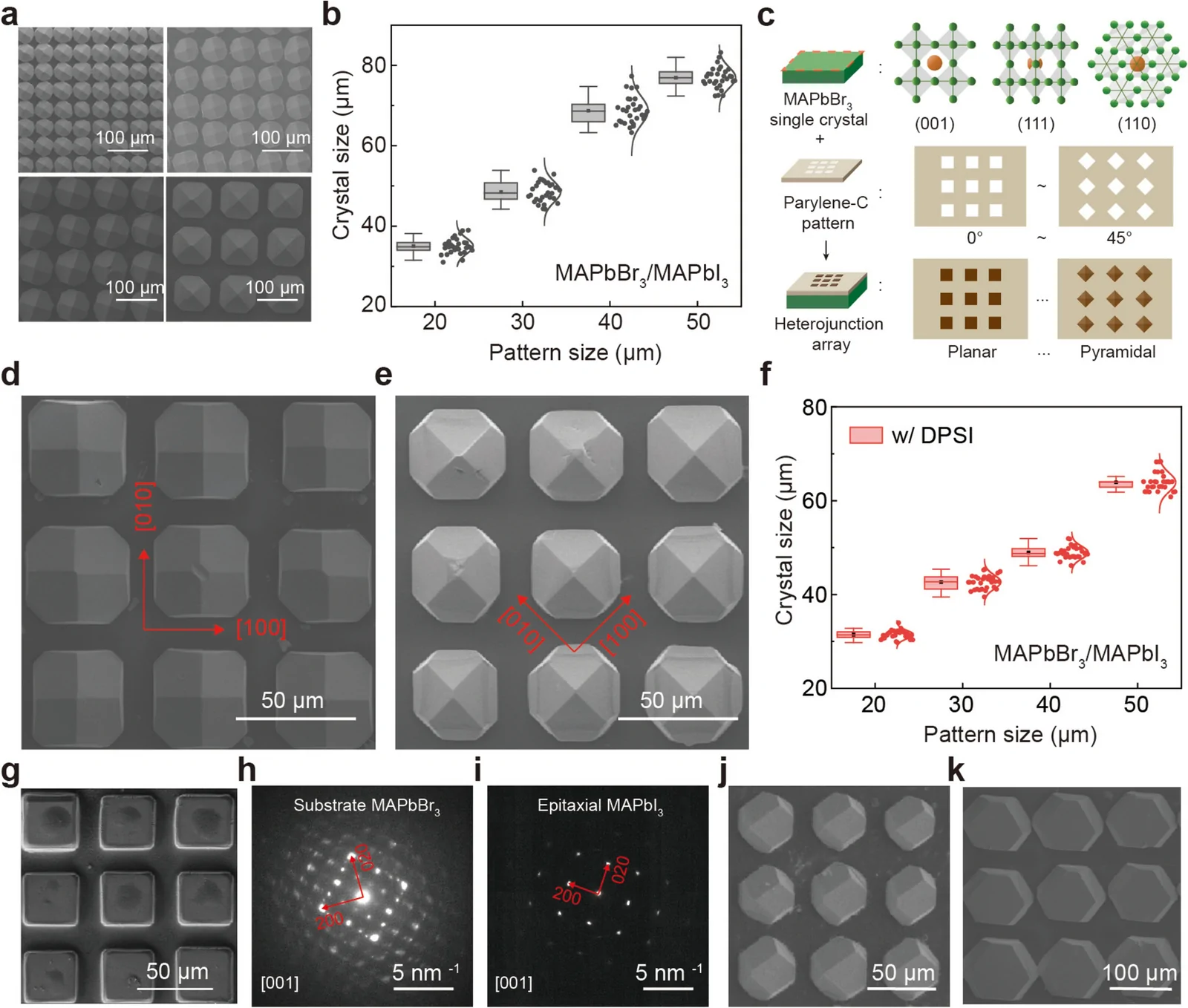
Controllable epitaxial growth of MAPbBr_3_/MAPbI_3_ perovskite single-crystal heterojunction arrays. **a** SEM images of selective epitaxial MAPbI_3_ single-crystal arrays with varying sizes. **b** Dependence of pixel size on template opening size for MAPbI_3_ single-crystal arrays epitaxially grown on MAPbBr_3_ substrates. **c** Schematic diagram of precise adjustment of pixel arrangement angle and crystal orientation by tuning the angle between pattern opening and substrate crystal orientation. SEM images of epitaxial MAPbI_3_ single-crystal arrays with different angles of **d** 0° and **e** 45°. **f** Pixel size versus template opening size for epitaxial MAPbI_3_ single-crystal arrays with the addition of DPSI to the MAPbBr_3_ precursor solution. **g** SEM image of epitaxial MAPbI_3_ perovskite single-crystal arrays with DPSI. **h** SAED patterns of the MAPbBr_3_ substrate. **i** SAED patterns of the corresponding epitaxial MAPbI_3_ perovskite single-crystal arrays with DPSI. SEM images of epitaxial MAPbI_3_ perovskite single-crystal arrays with different crystal orientations of **j** (111) and **k** (110)

The selective epitaxial growth method demonstrates versatile control over the geometric and crystallographic properties of the epitaxial perovskite arrays, including the precise regulation of the pixel dimensions, pixel arrangement angle and crystal orientation. The dimensions of the individual single-crystal pixels are highly dependent on the size of the openings in the Parylene-C film under a certain epitaxial growth condition. As shown in Fig. 3a, uniform MAPbI_3_ arrays with different predefined pixel dimensions were fabricated on the MAPbBr_3_ substrate. We statistically analyzed the dimensions of the 100 individual pixels for each Parylene-C opening size. The average width of the pixel exhibits a strong linear relationship with the Parylene-C opening size and the narrow width distribution for each set, indicating the excellent uniformity of the array (Fig. 3b). Furthermore, the width of the pixel is usually larger than the corresponding openings. For example, the openings of 30 µm generated pixels with an average size of about 45 µm, ensuring 100% coverage for each pixel. The rotational alignment of the entire array can be controlled by tuning the angle between the patterned openings and the crystallographic axes of the substrate (Fig. 3c). The MAPbI_3_ arrays with the openings rotated at 0° and 45° relative to the crystal directions of the substrate were fabricated. As shown in Fig. 3d, e the MAPbI_3_ pixels demonstrate a clear rotation with the angle of 45°, which precisely replicates the opening’s rotational alignment. In addition to the external geometric control, the crystal orientation of the array can be modified by introducing additives or using different substrate surfaces due to the lattice matching. The addition of DPSI to the precursor solution modifies the crystal habit of MAPbI_3_ arrays, promoting the exposure of the (100) facets rather than the (110) facets. The size control was also maintained during this process (Fig. 3f). Figure 3g shows the morphology of the epitaxial perovskite arrays with DPSI, transforming from the pyramidal shape to a cubic. Importantly, the SAED patterns of the substrate (Fig. 3h) and the epitaxial perovskite arrays with DPSI (Fig. 3i) show a consistent crystal orientation, confirming that the epitaxial relationship is preserved despite the change in crystal habit. Moreover, by utilizing MAPbBr_3_ substrates with different surface orientations, such as (111) and (110), both the morphologies and orientations of the MAPbI_3_ arrays can be directly changed correspondingly. As shown in Fig. 3j, k the pixel shapes changed to be consistent with the substrate orientations. This selective epitaxial growth method also demonstrated the controllability over the size, rotation and orientation of the MAPbBr_3_ arrays on the MAPbCl_3_ substrate (Figs. S6-S8), highlighting the capability of this method for controllable fabrication of single crystal perovskite arrays.

### Characterization of the Self-Powered Photodetectors Based on Perovskite Single-Crystal Heterojunction Arrays

**Fig. 4:**
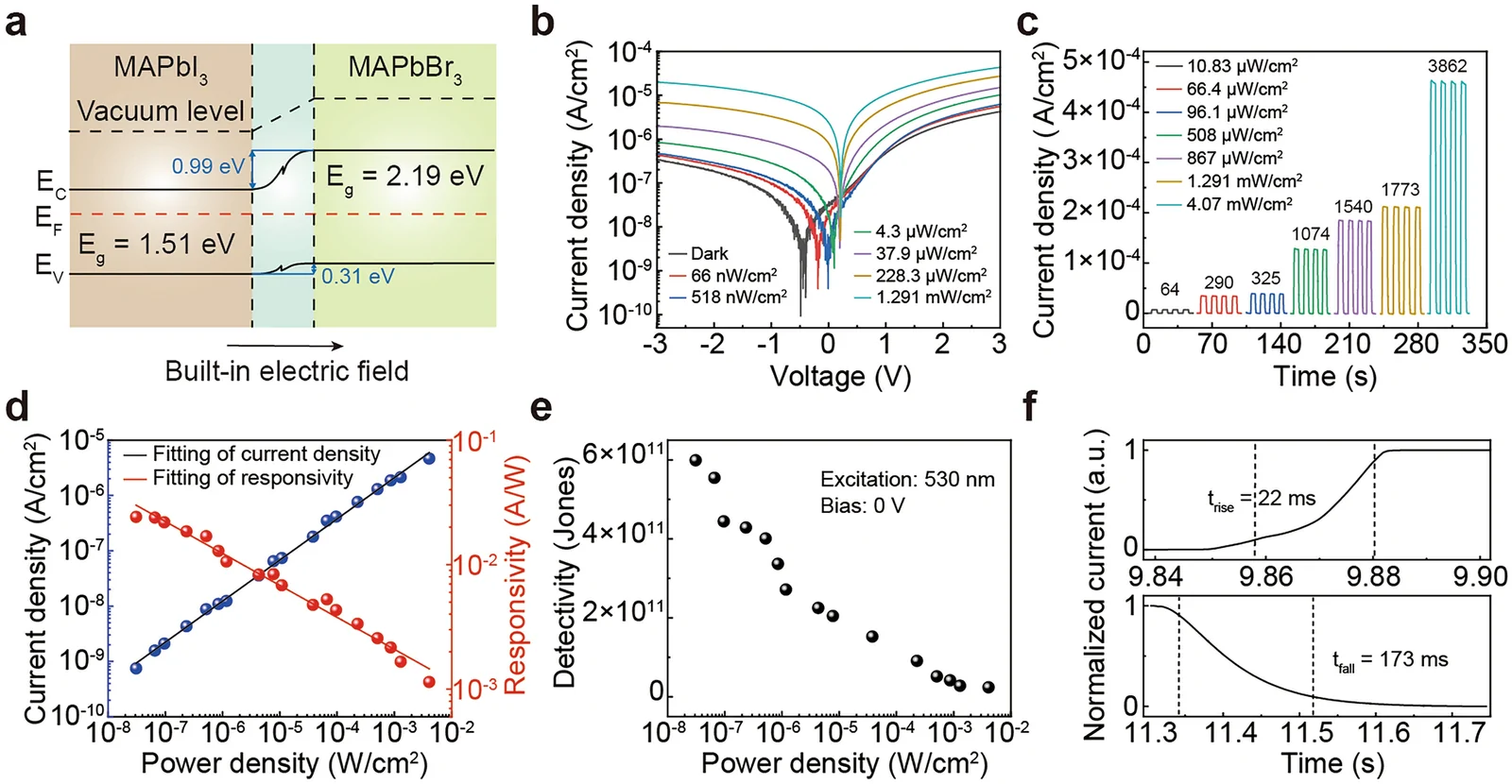
Optoelectronic response of the self-powered photodetector array based on MAPbBr_3_/MAPbI_3_ perovskite single-crystal heterojunction array. **a** Energy band diagram of the self-powered photodetector. **b** *J-V* characteristics under varying light intensities. **c** Current density of the device under different light irradiation. The specific value of the on–off ratio of the device is labeled on the curve. **d** Photocurrent density and responsivity under different light intensities. **e** Specific detectivity under different light intensities. **f** Response time of the self-powered photodetector array

Self-powered photodetector arrays (8 × 8 pixels) based on both MAPbBr_3_/MAPbI_3_ and MAPbCl_3_/MAPbBr_3_ heterojunction arrays were fabricated through the deposition of the patterned metal electrodes on the heterojunctions. Based on the absorption and ultraviolet photoelectron spectroscopy (UPS) spectra (Fig. S9), the valence band maximum (E_V_), Fermi level (E_F_), and conduction band minimum (E_C_) of the MAPbBr_3_/MAPbI_3_ heterojunction were calculated. The energy band diagram of the MAPbBr_3_/MAPbI_3_ photodetector is depicted in Fig. 4a, which forms a type-II heterojunction. This band structure creates a built-in electric field at the interface, which facilitates the efficient separation and transport of photogenerated electron–hole pairs, enabling the device operation at zero bias. This built-in electric field has been confirmed by the accelerated carrier extraction observed in TRPL (Fig. S10) and the distinct surface potential difference directly mapped via Kelvin probe force microscopy (KPFM) (Fig. S11). The current density–voltage (*J-V*) curves of the self-powered photodetector array under dark condition and 532 nm illumination with light intensity varying from 66 nW cm^−2^ to 1.291 mW cm^−2^ are shown in Fig. 4b. The normalized responsivity of the self-powered photodetector array under full spectrum is also characterized in the Fig. S12. Figure 4c shows the current density-time (*J-t*) characteristics of the device under 0 V bias. The device demonstrates stable and repeatable on/off switching behaviors across a wide range of light intensities. Importantly, the device maintains a stable photoresponse at an ultralow light intensity of 9 nW cm^−2^ (Fig. S13), demonstrating high sensitivity and a detection limit competitive with previously reported perovskite heterojunction photodetectors (Table S1). Responsivity under different illuminations is a key metric for assessing detector performance. Figure 4d plots the photocurrent density and responsivity as functions of light intensity. The photocurrent shows a linear dependence on the light intensity and the responsivity is higher at lower light intensities, exhibiting a peak responsivity of 2.4 mA W^−1^. The specific detectivity (D*) is plotted with the light intensity, reaching 6.0 × 10^11^ Jones under weak light conditions (Fig. 4e). The response time is defined as the time used for the current to switch between the 10% and 90% of its peak value. As shown in Fig. 4f, the self-powered photodetector array exhibits a fast response, with a rise time (*tᵣᵢₛₑ*) of 22 ms and a fall time (*t*_*fall*_) of 173 ms. Furthermore, the device exhibits excellent operational stability, exhibiting a 10% decay in photocurrent after 7000 s of continuous on/off switching (Fig. S14a-d). Long-term stability was also evaluated by measuring the optoelectronic response after storage in ambient air for four weeks without encapsulation. As shown in Fig. S14e and f, the device retains stable switching behavior during the four weeks of ambient exposure, exhibiting a 10% degradation over this extended period. The optoelectronic performance of the self-powered photodetector arrays based on the MAPbCl_3_/MAPbBr_3_ heterojunction is summarized in Fig. S15. These results demonstrate that epitaxially grown single-crystal heterojunction arrays serve as a promising structure for the self-powered photodetectors. While the optoelectronic anisotropy of bulk perovskite single crystals has been investigated [54–60], establishing a systematic quantitative correlation between specific crystal orientations (e.g., (100), (110), (111)) and device performance remains challenging in these heterojunction arrays due to geometric and electrode contact constraints, which could be an important direction for future structure–property relationship investigations.

**Fig. 5:**
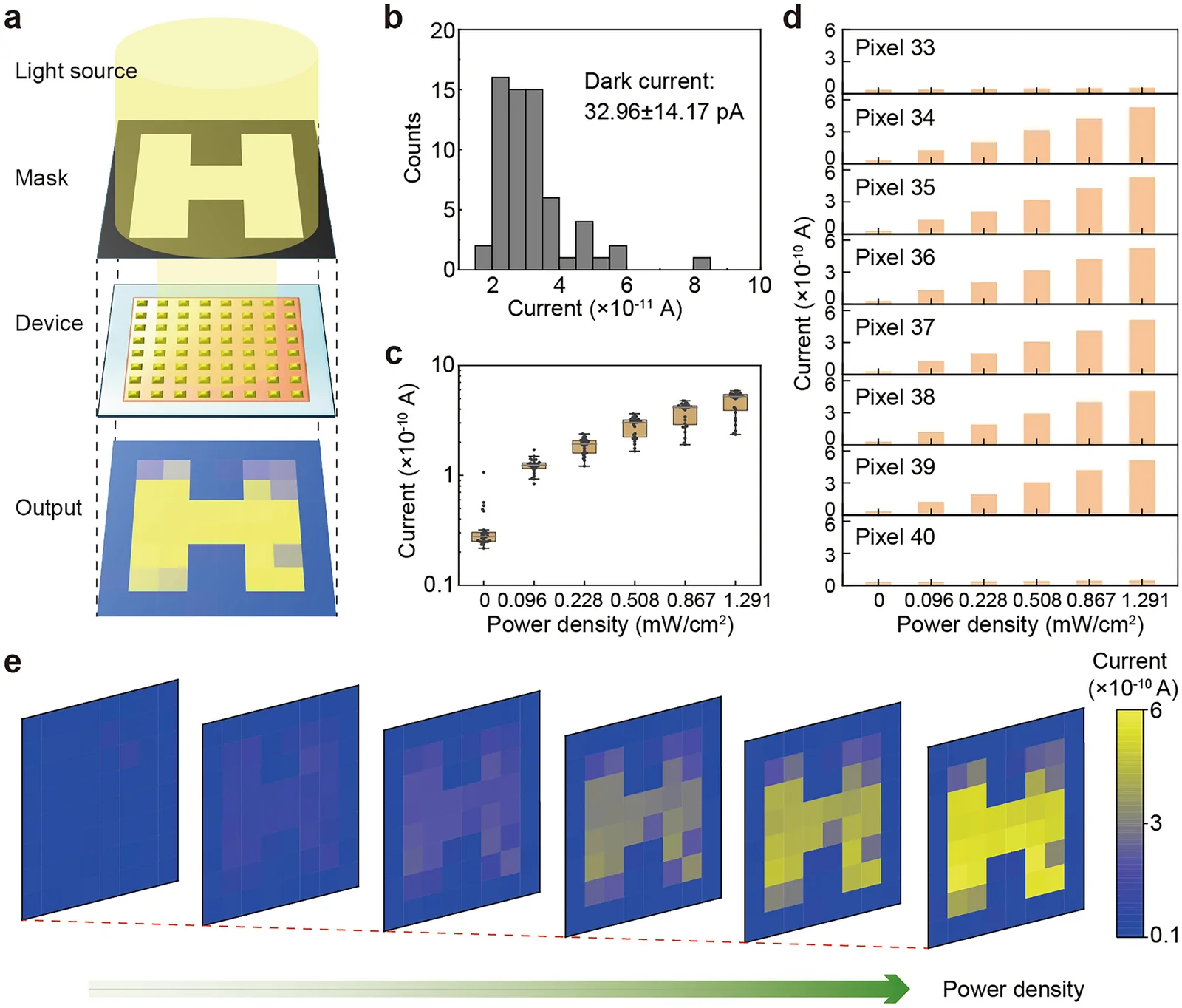
Imaging applications of the self-powered photodetector array based on MAPbBr_3_/MAPbI_3_ perovskite single-crystal heterojunction array. **a** Schematic of the imaging process using the self-powered photodetector array. **b** Statistical distribution of dark current for all 64 pixels within the 8 × 8 array. **c** Statistical distribution of photocurrent for all 64 pixels under illumination at different light intensities. **d** Output current of eight pixels (Pixels 33–40) along the fifth row of the array device under varying light intensities. **e** Photocurrent mapping of the 8 × 8 array acquired under the different light intensities

We further investigated the imaging capability of the 8 × 8 self-powered photodetector array. The imaging process is shown in Fig. 5a. In this setup, a light source projects an optical pattern through a mask onto the 8 × 8 device array, and an image is reconstructed by mapping the individual photocurrent response of each pixel. High pixel-to-pixel uniformity in both dark and illuminated conditions is important for the imaging applications. We first evaluated the dark current distribution across all 64 pixels. As shown in Fig. 5b, the dark current of all the pixels exhibits a concentrated distribution with a low average dark current of 32.96 ± 14.17 pA. Figure 5c demonstrates photocurrent distribution under various light intensities. With the light intensity increasing, the photocurrents of pixels enhanced accordingly while maintaining a narrow distribution, indicating a consistent and uniform photoresponse across the entire array. The ability to spatially resolve light patterns with minimal electrical crosstalk is also critical for a photodetector array. We selectively illuminated the central six pixels (pixels 34–39) of a single row while keeping the adjacent pixels (pixel 33 and 40) in the dark. As shown in Figs. 5d and S16, the currents of the illuminated pixels increase obviously with light intensity, while the currents of the dark pixel 33 and pixel 40 remaining unchanged. This result confirms that crosstalk between adjacent pixels is negligible, despite using the discrete heterojunction arrays on the continuous substrate. An 'H'-shaped light pattern was then projected onto the photodetector array. Figure 5e shows the resulting two-dimensional photocurrent maps recorded at different light intensities. As the light intensity increases, the 'H' pattern becomes clear. Specifically, as the illumination intensity gradually increases from 0 to 1.291 mW cm^−2^, the photocurrent mapping of the input pattern exhibits a continuous multi-level response. Furthermore, to evaluate the imaging capabilities under complex scenarios, we scaled up the device to a 16 × 16 MAPbBr_3_/MAPbI_3_ perovskite single-crystal heterojunction array (Fig. S17). A “Christmas tree” pattern was projected onto the array. The photocurrent mapping accurately reconstructed the sharp geometric outlines and spatial features of the pattern. By extracting the modulation transfer function (MTF), the device demonstrated a spatial resolution of 8.0 lp mm^−1^ at an MTF of 0.2, confirming the capability for resolving fine optical details.

## Conclusions

In summary, we demonstrated a simple and versatile strategy for the fabrication of orientation-controlled single-crystal perovskite heterojunction arrays. By combining the patterned Parylene-C film for spatial confinement with the single-crystal substrate for epitaxial guidance, this method demonstrated the precise control over the geometric parameters and crystallographic properties of the array. The resulting MAPbCl_3_/MAPbBr_3_ and MAPbBr_3_/MAPbI_3_ heterojunction arrays exhibited high crystal quality and uniformity, which enabled high sensitivity and imaging capability of the corresponding self-powered photodetector arrays based on these heterojunctions.

## Supplementary Information

Below is the link to the electronic supplementary material.

Supplementary file1 (DOCX 3868 KB)

## References

[1] L. Zhang, M. Zhang, H. Wang, Z. Li, Z. Zhang et al., Diverse perovskite solar cells: progress, challenges, and perspectives. Adv. Mater. **38**(1), e12221 (2026). 10.1002/adma.202512221 40923487 10.1002/adma.202512221

[2] S. Jia, C. Gu, X. Zhou, Y. Miao, Y. Tian et al., Self-assembled monolayers for high-performance perovskite solar cells. Adv. Funct. Mater. **36**(12), e12747 (2026). 10.1002/adfm.202512747

[3] S.A. Ali Shah, M.H. Sayyad, Z. Guo, Light-emitting perovskite solar cells: genesis to recent drifts. Solar RRL **8**(23), 2400652 (2024). 10.1002/solr.202400652

[4] Y.-H. Lin, Vikram, F. Yang, X.-L. Cao, A. Dasgupta et al., Bandgap-universal passivation enables stable perovskite solar cells with low photovoltage loss. Science **384**(6697), 767–775 (2024).10.1126/science.ado2302 10.1126/science.ado2302 38753792

[5] D. Gao, B. Li, Q. Liu, C. Zhang, Z. Yu et al., Long-term stability in perovskite solar cells through atomic layer deposition of tin oxide. Sci **386**(6718), 187–192 (2024). 10.1126/science.adq8385 10.1126/science.adq8385 39388552

[6] Q. Zhou, G. Huang, J. Wang, T. Miao, R. Chen et al., Aromatic interaction-driven out-of-plane orientation for inverted perovskite solar cells with improved efficiency. Nat. Energy **10**(11), 1371–1381 (2025). 10.1038/s41560-025-01882-x

[7] Y. Li, X. Guan, Y. Zhao, Q. Zhang, X. Chen et al., Modulation of charge transport layer for perovskite light-emitting diodes. Adv. Mater. **37**(25), 2410535 (2025). 10.1002/adma.202410535 10.1002/adma.202410535 39443833

[8] H. Liu, G. Shi, C. Peng, W. Chen, H. Yao et al., Advances and challenges in large-area perovskite light-emitting diodes. Adv. Mater. **37**(25), e2410154 (2025). 10.1002/adma.202410154 39318091 10.1002/adma.202410154

[9] Y. Deng, J. Liu, W. Liu, Y. Zhang, H. Gao et al., Buried interface modification for high-performance perovskite light-emitting diodes. Adv. Opt. Mater. **13**(26), e00894 (2025). 10.1002/adom.202500894

[10] S. Zheng, Z. Wang, N. Jiang, H. Huang, X. Wu et al., Ultralow voltage–driven efficient and stable perovskite light-emitting diodes. Sci. Adv. **10**(36), eadp8473 (2024). 10.1126/sciadv.adp8473 39241067 10.1126/sciadv.adp8473 PMC11378915

[11] Q. Zhang, K. Yang, C. Luo, Z. Lin, W. Chen et al., Nanosecond response perovskite quantum dot light-emitting diodes with ultra-high resolution for active display application. Light Sci. Appl. **14**(1), 285 (2025). 10.1038/s41377-025-01959-y 40841515 10.1038/s41377-025-01959-y PMC12370927

[12] Y. Sun, L. Ge, L. Dai, C. Cho, J. Ferrer Orri et al., Bright and stable perovskite light-emitting diodes in the near-infrared range. Nat. **615**(7954), 830–835 (2023). 10.1038/s41586-023-05792-4 10.1038/s41586-023-05792-4 36922588

[13] Z. Xu, X. Han, W. Wu, F. Li, R. Wang et al., Controlled on-chip fabrication of large-scale perovskite single crystal arrays for high-performance laser and photodetector integration. Light Sci. Appl. **12**(1), 67 (2023). 10.1038/s41377-023-01107-4 36882401 10.1038/s41377-023-01107-4 PMC9992671

[14] J. Meng, Q. Li, J. Huang, C. Pan, Z. Li, Self-powered photodetector for ultralow power density UV sensing. Nano Today **43**, 101399 (2022). 10.1016/j.nantod.2022.101399

[15] W. Wu, H. Lu, X. Han, C. Wang, Z. Xu et al., Recent progress on wavelength-selective perovskite photodetectors for image sensing. Small Methods **7**(4), e2201499 (2023). 10.1002/smtd.202201499 36811238 10.1002/smtd.202201499

[16] W. Wang, W. Tian, F. Chen, J. Wang, W. Zhai et al., Filter-less color-selective photodetector derived from integration of parallel perovskite photoelectric response units. Adv. Mater. **36**(33), 2404968 (2024). 10.1002/adma.202404968 10.1002/adma.202404968 38897182

[17] Z. Zhu, H. Chen, W. Huang, B. Zhao, S. Gao et al., Ion leakage current control for polycrystalline metal halide perovskite direct X-ray detectors. ACS Appl. Mater. Interfaces **16**(39), 53177–53185 (2024). 10.1021/acsami.4c10707 39295274 10.1021/acsami.4c10707

[18] L. Min, H. Sun, L. Guo, M. Wang, F. Cao et al., Frequency-selective perovskite photodetector for anti-interference optical communications. Nat. Commun. **15**(1), 2066 (2024). 10.1038/s41467-024-46468-5 38453948 10.1038/s41467-024-46468-5 PMC10920912

[19] X. Han, J. Tao, Y. Liang, F. Guo, Z. Xu et al., Ultraweak light-modulated heterostructure with bidirectional photoresponse for static and dynamic image perception. Nat. Commun. **15**(1), 10430 (2024). 10.1038/s41467-024-54845-3 39616156 10.1038/s41467-024-54845-3 PMC11608218

[20] Z. He, B. Sun, H. Lu, X. Sun, Z. Xu et al., Focus-tunable real-time imaging system based on ultrathin perovskite curved image sensor with hierarchical mesh design. Sci. Adv. **11**(36), eadw7826 (2025). 10.1126/sciadv.adw7826 40901939 10.1126/sciadv.adw7826 PMC12407062

[21] T.M.H. Nguyen, S.G. Shin, H.W. Choi, C.W. Bark, Recent advances in self-powered and flexible UVC photodetectors. Explor. **2**(5), 20210078 (2022). 10.1002/EXP.20210078 10.1002/EXP.20210078 PMC10190973 37325501

[22] X. Cao, S. Xing, R. Lai, Y. Lian, Y. Wang et al., Low-threshold, external-cavity-free flexible perovskite lasers. Adv. Funct. Mater. **33**(19), 2211841 (2023). 10.1002/adfm.202211841

[23] J. Moon, Y. Mehta, K. Gundogdu, F. So, Q. Gu, Metal-halide perovskite lasers: cavity formation and emission characteristics. Adv. Mater. **36**(20), 2211284 (2024). 10.1002/adma.202211284 10.1002/adma.202211284 36841548

[24] Y. Li, S. Liu, T. Feeney, J. Roger, M. Gholipoor et al., Electrically-switchable gain in optically pumped CsPbBr_3_ lasers with low threshold at nanosecond pumping. Small **21**(13), 2411935 (2025). 10.1002/smll.202411935 39989085 10.1002/smll.202411935 PMC11962692

[25] C. Zou, X. Cao, Z. Wang, Y. Yang, Y. Lian et al., Continuous-wave perovskite polariton lasers. Sci. Adv. **11**(2), eadr8826 (2025). 10.1126/sciadv.adr8826 39792669 10.1126/sciadv.adr8826 PMC11721563

[26] X. Li, Y. Jia, M. Liu, S. He, J. Guo et al., Ultrastable lasing from perovskite colloidal nanocrystals. Sci. Adv. **11**(28), eadq9002 (2025). 10.1126/sciadv.adq9002 40632855 10.1126/sciadv.adq9002 PMC12239972

[27] C. Ge, Y. Li, H. Song, Q. Xie, L. Zhang et al., Anisotropic carrier dynamics and laser-fabricated luminescent patterns on oriented single-crystal perovskite wafers. Nat. Commun. **15**(1), 914 (2024). 10.1038/s41467-024-45055-y 38291033 10.1038/s41467-024-45055-y PMC10828488

[28] G. Vats, B. Hodges, A.J. Ferguson, L.M. Wheeler, J.L. Blackburn, Optical memory, switching, and neuromorphic functionality in metal halide perovskite materials and devices. Adv. Mater. **35**(37), 2205459 (2023). 10.1002/adma.202205459 10.1002/adma.202205459 36120918

[29] S. Liu, P. Li, X. Fu, K. Zhou, Y. Ji et al., Ionic behaviors of perovskite devices and their neuromorphic applications. Adv. Funct. Mater. **36**(15), e10934 (2026). 10.1002/adfm.202510934

[30] T. Liu, H. Wang, C. Sun, Z. Yuan, X. Wang et al., Suppression of tin oxidation *via* Sn→B bonding interactions for high-resolution lead-free perovskite neuromorphic imaging sensors. Adv. Mater. **37**(29), 2502015 (2025). 10.1002/adma.202502015 10.1002/adma.202502015 40341612

[31] R.A. John, A. Milozzi, S. Tsarev, R. Brönnimann, S.C. Boehme et al., Ionic-electronic halide perovskite memdiodes enabling neuromorphic computing with a second-order complexity. Sci. Adv. **8**(51), eade0072 (2022). 10.1126/sciadv.ade0072 36563153 10.1126/sciadv.ade0072 PMC9788778

[32] H. Wang, B. Sun, S.S. Ge, J. Su, M.L. Jin, On non-von Neumann flexible neuromorphic vision sensors. npj Flex. Electron. **8**, 28 (2024). 10.1038/s41528-024-00313-3

[33] Z. Yang, S. Huo, Z. Zhang, F. Meng, B. Liu et al., High-precision multibit opto-electronic synapses based on ReS_2_/h-BN/graphene heterostructure for energy-efficient and high-accuracy neuromorphic computing. Adv. Funct. Mater. **35**(48), 2509119 (2025). 10.1002/adfm.202509119

[34] F. Guo, X. Yang, P. Wang, X. Bai, T. Kong et al., Advances in single-crystal films: Synergistic insights from perovskites and organic molecules for high-performance optoelectronics. Small **21**(19), 2412101 (2025). 10.1002/smll.202412101 10.1002/smll.202412101 39989101

[35] W. Wu, X. Han, J. Li, X. Wang, Y. Zhang et al., Ultrathin and conformable lead halide perovskite photodetector arrays for potential application in retina-like vision sensing. Adv. Mater. **33**(9), 2006006 (2021). 10.1002/adma.202006006 10.1002/adma.202006006 33475208

[36] Q. Wang, G. Zhang, H. Zhang, Y. Duan, Z. Yin et al., High-resolution, flexible, and full-color perovskite image photodetector *via* electrohydrodynamic printing of ionic-liquid-based ink. Adv. Funct. Mater. **31**(28), 2100857 (2021). 10.1002/adfm.202100857

[37] Y. Chen, C. Zhao, T. Zhang, X. Wu, W. Zhang et al., Flexible and filter-free color-imaging sensors with multicomponent perovskites deposited using enhanced vapor technology. Small **17**(26), 2007543 (2021). 10.1002/smll.202007543 10.1002/smll.202007543 34096175

[38] B. Peng, H. Zhou, Z. Liu, Y. Li, Q. Shang et al., Pattern-selective molecular epitaxial growth of single-crystalline perovskite arrays toward ultrasensitive and ultrafast photodetector. Nano Lett. **22**(7), 2948–2955 (2022). 10.1021/acs.nanolett.2c00074 35289627 10.1021/acs.nanolett.2c00074

[39] Y. Hou, J. Li, J. Yoon, A.M. Knoepfel, D. Yang et al., Retina-inspired narrowband perovskite sensor array for panchromatic imaging. Sci. Adv. **9**(15), eade2338 (2023). 10.1126/sciadv.ade2338 37058567 10.1126/sciadv.ade2338 PMC10104461

[40] Z. Long, X. Qiu, C.L.J. Chan, Z. Sun, Z. Yuan et al., A neuromorphic bionic eye with filter-free color vision using hemispherical perovskite nanowire array retina. Nat. Commun. **14**(1), 1972 (2023). 10.1038/s41467-023-37581-y 37031227 10.1038/s41467-023-37581-y PMC10082761

[41] Y. Zhou, D. Liu, H.G. Yang, S. Yang, Y. Hou, Preparation techniques for perovskite single crystal films: from nucleation to growth. Chem. **20**(4), e202401294 (2025). 10.1002/asia.202401294 10.1002/asia.202401294 39624991

[42] Y. Pan, X. Wang, Y. Liao, Y. Xu, Y. Li et al., Epitaxial perovskite single-crystalline heterojunctions for filter-free ultra-narrowband detection with tunable spectral responses. ACS Appl. Mater. Interfaces **14**(44), 50331–50342 (2022). 10.1021/acsami.2c13126 36300824 10.1021/acsami.2c13126

[43] Y. He, S. Gao, B. Zhao, H. Chen, Z. Zhu et al., Epitaxial growth of high-quality perovskite heterojunctions for direct-conversion X-ray detectors. ACS Energy Lett. **10**(6), 2770–2777 (2025). 10.1021/acsenergylett.5c00938

[44] Y. Guan, C. Zhang, Z. Liu, Y. Zhao, A. Ren et al., Single-crystalline perovskite p–n junction nanowire arrays for ultrasensitive photodetection. Adv. Mater. **34**(35), 2203201 (2022). 10.1002/adma.202203201 10.1002/adma.202203201 35801692

[45] J. Zhang, C. Li, Y. Liang, J. Song, Q. Shang et al., Solution-processed selective area homoepitaxial growth of suspended MAPbX_3_ (X = Cl, Br) perovskite micro-arrays. Adv. Funct. Mater. **33**(4), 2208841 (2023). 10.1002/adfm.202208841

[46] J. Yan, F. Gao, Y. Tian, Y. Li, W. Gong et al., Controllable perovskite single crystal heterojunction for stable self-powered photo-imaging and X-ray detection. Adv. Opt. Mater. **10**(17), 2200449 (2022). 10.1002/adom.202200449

[47] Y. Peng, J. Lu, X. Wang, W. Ma, M. Que et al., Self-powered high-performance flexible GaN/ZnO heterostructure UV photodetectors with piezo-phototronic effect enhanced photoresponse. Nano Energy **94**, 106945 (2022). 10.1016/j.nanoen.2022.106945

[48] Y. Chen, X. Peng, W. Qin, S. Li, L. Zhang et al., Filterless bandpass photodetectors enabled by 2D/3D perovskite heterojunctions. Adv. Funct. Mater. **34**(41), 2403942 (2024). 10.1002/adfm.202403942

[49] Z. Guo, J. Zhang, X. Liu, L. Wang, L. Xiong et al., Optoelectronic synapses and photodetectors based on organic semiconductor/halide perovskite heterojunctions: materials, devices, and applications. Adv. Funct. Mater. **33**(46), 2305508 (2023). 10.1002/adfm.202305508

[50] J. Hao, Y.-H. Kim, S.N. Habisreutinger, S.P. Harvey, E.M. Miller et al., Low-energy room-temperature optical switching in mixed-dimensionality nanoscale perovskite heterojunctions. Sci. Adv. **7**(18), eabf1959 (2021). 10.1126/sciadv.abf1959 33910894 10.1126/sciadv.abf1959 PMC8081365

[51] X. Li, W. Zhang, X. Guo, C. Lu, J. Wei et al., Constructing heterojunctions by surface sulfidation for efficient inverted perovskite solar cells. Sci. **375**(6579), 434–437 (2022). 10.1126/science.abl5676 10.1126/science.abl5676 35084976

[52] D. Liu, J. Bi, W. Xu, K.W.P. Orr, F. Wang et al., Strain relaxation in halide perovskites *via* 2D/3D perovskite heterojunction formation. Sci. Adv. **11**(26), eadu3459 (2025). 10.1126/sciadv.adu3459 40577478 10.1126/sciadv.adu3459 PMC12204152

[53] B. Li, Q. Liu, J. Gong, S. Li, C. Zhang et al., Harnessing strong aromatic conjugation in low-dimensional perovskite heterojunctions for high-performance photovoltaic devices. Nat. Commun. **15**(1), 2753 (2024). 10.1038/s41467-024-47112-y 38553436 10.1038/s41467-024-47112-y PMC10980693

[54] L. Zhang, S. Cui, Q. Guo, C. Ge, Q. Han et al., Anisotropic performance of high-quality MAPbBr_3_ single-crystal wafers. ACS Appl. Mater. Interfaces **12**(46), 51616–51627 (2020). 10.1021/acsami.0c14582 33164486 10.1021/acsami.0c14582

[55] P. Zhang, G. Zhang, L. Liu, D. Ju, L. Zhang et al., Anisotropic optoelectronic properties of melt-grown bulk CsPbBr_3_ single crystal. J. Phys. Chem. Lett. **9**(17), 5040–5046 (2018). 10.1021/acs.jpclett.8b01945 30102540 10.1021/acs.jpclett.8b01945

[56] P. Saha, M.M. Rahman, C.L. Tolbert, C.M. Hill, Facet-dependent photoelectrochemistry on single crystal organic-inorganic halide perovskite electrodes. Chem. Biomed. Imaging **1**(5), 488–494 (2023). 10.1021/cbmi.3c00069 37655168 10.1021/cbmi.3c00069 PMC10467489

[57] S. Dong, Z.-Y. Hu, P. Wei, J. Han, Z. Wang et al., All-inorganic perovskite single-crystal photoelectric anisotropy. Adv. Mater. **34**(37), 2204342 (2022). 10.1002/adma.202204342 10.1002/adma.202204342 35891614

[58] Z.Y. Zhang, G.P. Wang, High-performance photoconductive unidirectional rectification in perovskite single-crystal. Adv. Opt. Mater. **14**(10), e03555 (2026). 10.1002/adom.202503555

[59] X. Cheng, L. Jing, Y. Zhao, S. Du, J. Ding et al., Crystal orientation-dependent optoelectronic properties of MAPbCl_3_ single crystals. J. Mater. Chem. C **6**(6), 1579–1586 (2018). 10.1039/c7tc05156e

[60] N.K. Tailor, S. Satapathi, Anisotropy in perovskite single crystals: from fundamentals to applications. J. Phys. Chem. C **126**(42), 17789–17803 (2022). 10.1021/acs.jpcc.2c05534

