# Supplementary material for: Controlled Epitaxial Growth of Perovskite Single-Crystal Heterojunction Arrays for Self-Powered Imaging
Source: Nanomicro Lett. 2026 May 29;18:391. doi: 10.1007/s40820-026-02224-6 (PMC13221491; doi:10.1007/s40820-026-02224-6)
Supplement: Supplementary file 1 — Supplementary file1 (DOCX 3868 KB) [file 40820_2026_2224_MOESM1_ESM.docx]

Supporting Information for

**Controlled Epitaxial Growth of** **Perovskite Single-Crystal** **Heterojunction Arrays for Self-powered Imaging**

Hui Lu^1,2,3#^, Yang Yu^1#^, Wenqiang Wu^2*^, Zeping He^1,2,3^, Kaiyu Hu^2^, Wenqiang Yang^2^, Xun Han^2,4*^, Caofeng Pan^1,2,3*^

^1^ CAS Center for Excellence in Nanoscience, Beijing Key Laboratory of Micro-nano Energy and Sensor, Beijing Institute of Nanoenergy and Nanosystems, Chinese Academy of Sciences, Beijing 101400, P. R. China

^2^ Institute of Atomic Manufacturing, Beihang University, Beijing 100191, P. R. China

^3^ School of Nanoscience and Technology, University of Chinese Academy of Sciences, Beijing 100049, P. R. China

^4^ Department of Applied Physics, The Hong Kong Polytechnic University, Hong Kong 999077, P. R. China

#Hui Lu and Yang Yu contributed equally to this work.

*Corresponding authors. Email: [wuwenqiang@buaa.edu.cn](mailto:wuwenqiang@buaa.edu.cn) (Wenqiang Wu); xunhan@buaa.edu.cn (Xun Han); [pancaofeng@buaa.edu.cn](mailto:pancaofeng@buaa.edu.cn) (Caofeng Pan)

**Supplementary Figures and Tables**


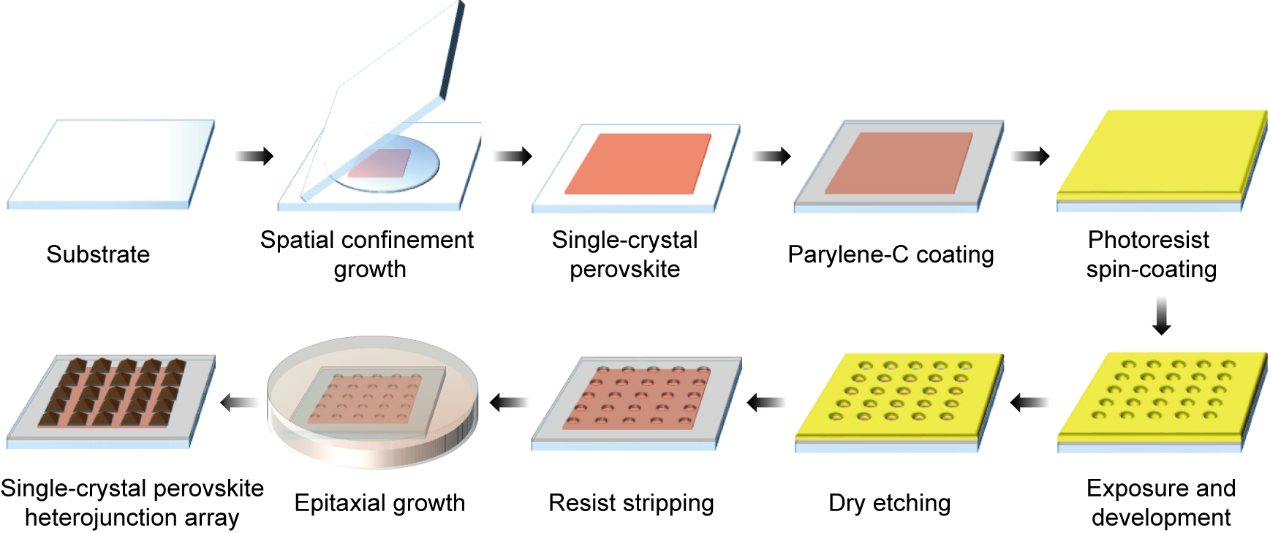


**Fig. S1** Schematic illustration of the detailed fabrication process for the patterned perovskite single-crystal heterojunction arrays


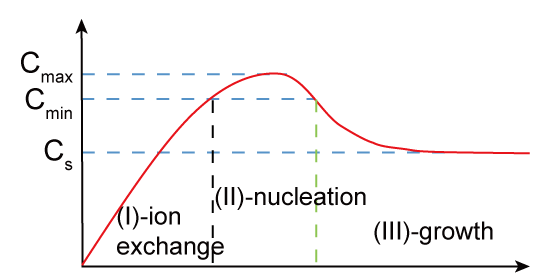


**Fig. S2** The mechanism of epitaxial growth of perovskite single-crystal heterojunction arrays. Stage I: Ion Exchange. Halogens (Cl⁻, Br⁻, I⁻) in halide perovskites exhibit extremely high mobility and diffusion coefficients even at room temperature. When the precursor solution of the epitaxial layer contacts the substrate, rapid inter-diffusion of halide ions occurs at the interface, rather than direct growth. This process forms a compositionally graded interfacial layer, during which the formation of the graded junction takes place. This graded layer effectively relieves stress induced by lattice mismatch, laying the foundation for subsequent high-quality epitaxial growth. Stage II: Nucleation. During nucleation, the epitaxial layer grows along the crystal orientation that minimizes lattice mismatch and interfacial energy at the surface. This is the most critical stage determining the crystal orientation and interfacial quality of the epitaxial layer. Stage III: Growth. Following nucleation, the crystal domains grow laterally (parallel to the interface) and vertically (perpendicular to the interface) under continuous precursor supply. If the lateral growth rate significantly exceeds the vertical rate, a continuous 2D thin film tends to form. Conversely, if vertical growth dominates and nucleation sites are confined, 3D single-crystal arrays are more likely to form.


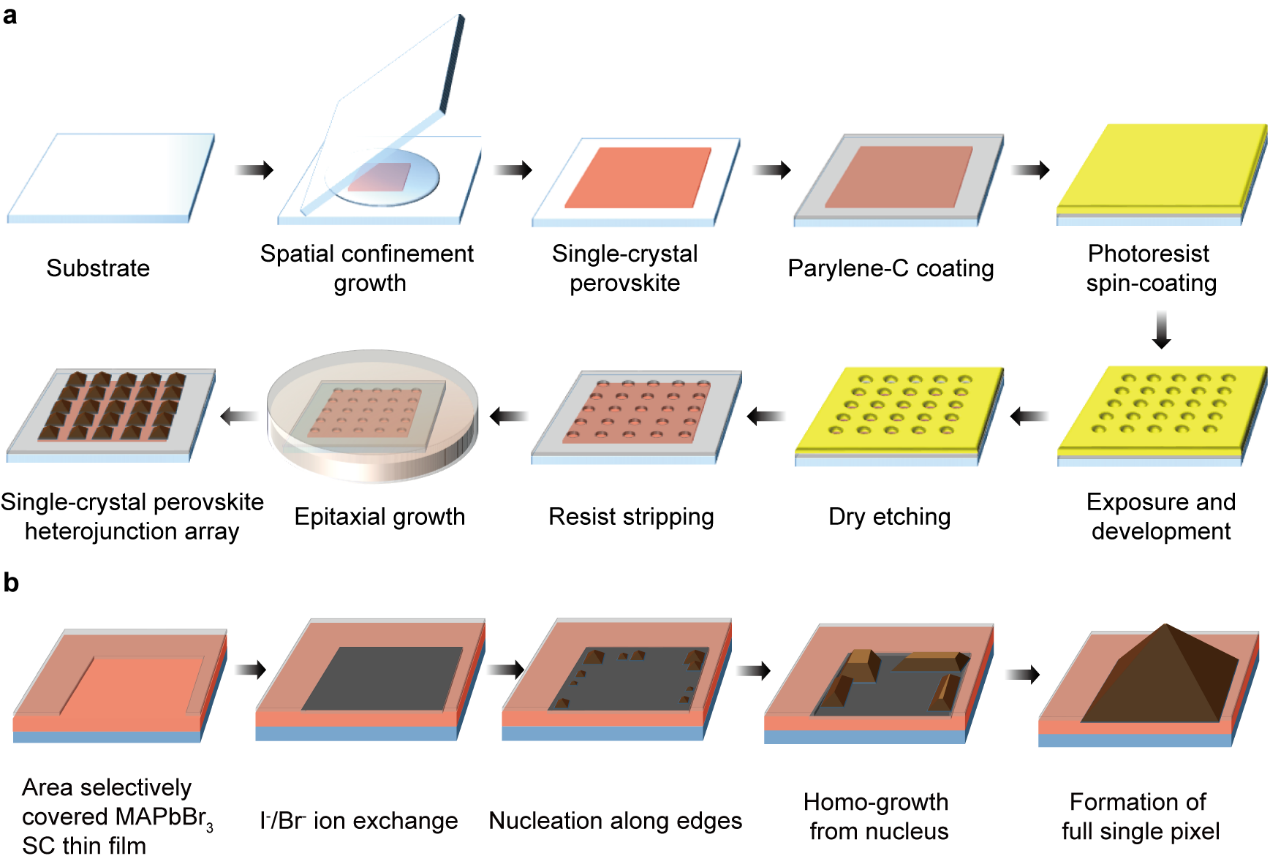


**Fig. S3** Schematic diagram of single-crystal perovskite heterojunction growth process. Initiated by an I^-^/Br^-^ ion exchange process on a pre-patterned MAPbBr_3_ thin film, the edges of the film are engineered to serve as preferential nucleation sites. The new perovskite phase then grows homoepitaxially from these edge nuclei, finally coalescing into a fully formed single-pixel heterojunction


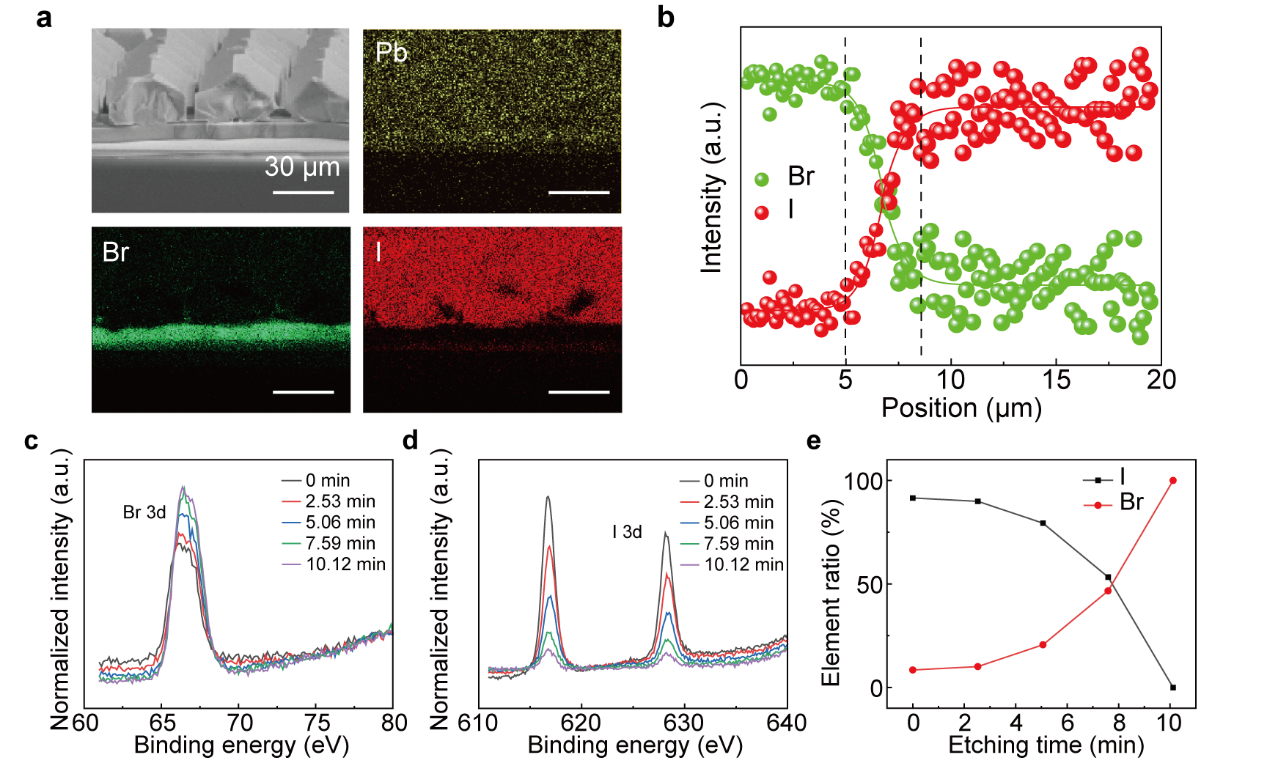


**Fig. S4** **a** EDS map of the MAPbBr_3_/MAPbI_3_ single-crystal heterojunction. **b** The distribution of halogen elements across the MAPbBr_3_/MAPbI_3_ single-crystal heterojunction. **c, d** Evolution of Br 3d and I 3d XPS spectra with etching time. **e** The quantified percentage of two halide ions obtained from XPS spectra at different etching time. With increasing Ar^+^ etching time, the intensity of the I 3d peak gradually decreases, while that of the Br 3d peak gradually increases. As a result, the extracted element ratio profile transitions from an I-rich state to a Br-rich state, confirming the gradient heterojunction structure


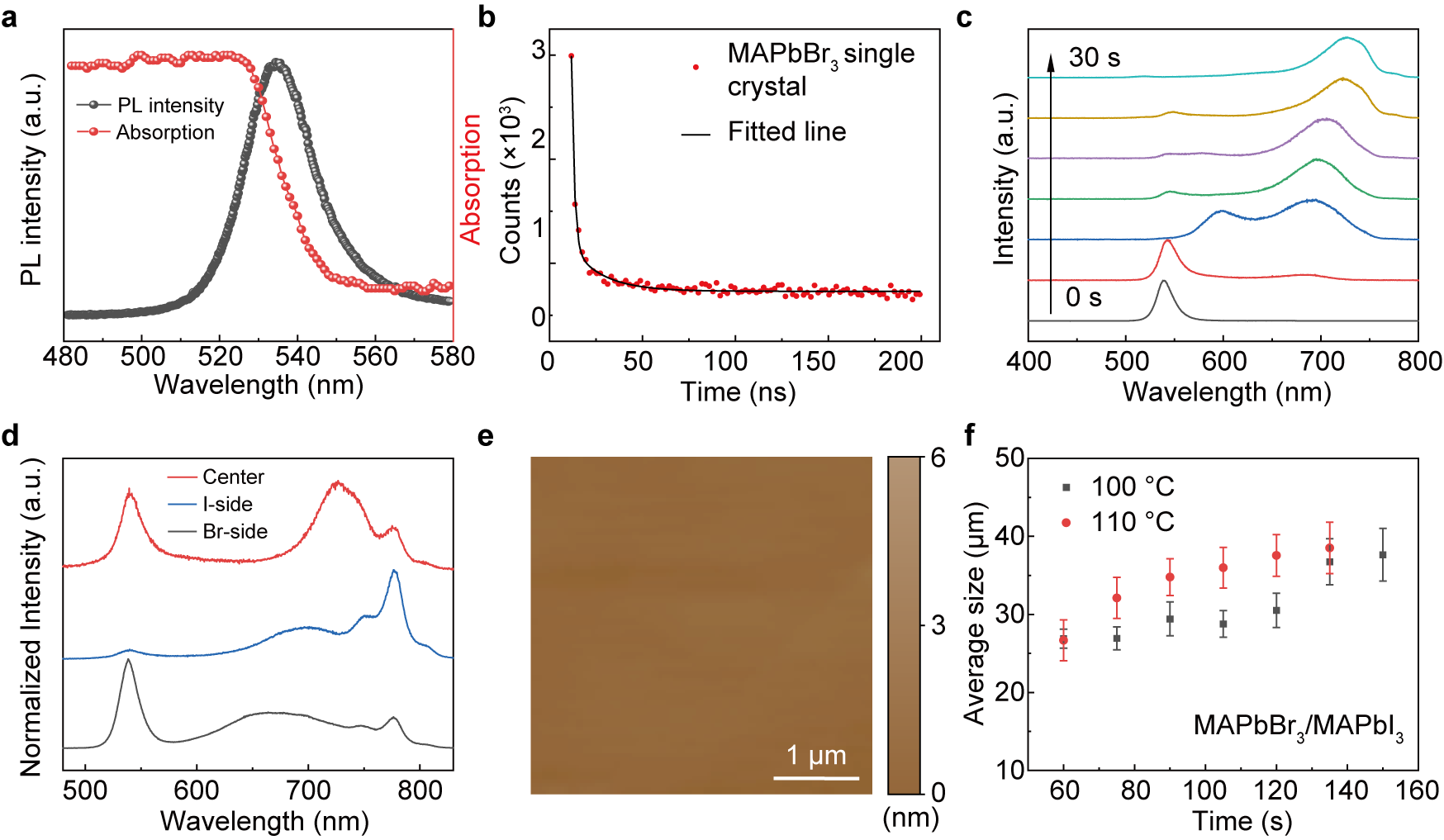


**Fig. S5** Performance characterization of MAPbBr_3_/MAPbI_3_ perovskite single-crystal heterojunction arrays. **a** PL and absorption spectra of a MAPbBr_3_ single crystal, showing a PL peak located at 535 nm and an absorption edge at 2.26 eV. **b** TRPL decay curve of the MAPbBr_3_ single crystal, fitted with a bi-exponential function yielding fast and slow decay times of 1.56 ns and 16.74 ns, respectively, affirming its high crystalline quality. **c** Evolution of PL spectra with growth duration. The PL peak exhibits a systematic red-shift from 0 to 30 s, indicating the formation of mixed-halide perovskites with progressively increasing I content via anion exchange. **d** Spatially resolved PL spectra across the MAPbBr_3_/MAPbI_3_ heterojunction. The absence of trap-state-related emission throughout the junction region suggests a negligible density of optically active defects. **e** AFM height image of the epitaxial MAPbI_3_ single crystal. The measured root-mean-square (RMS) roughness of 4.483 nm over a 20 × 20 μm^2^ area confirms its smooth surface morphology. **f** Correlation of average array pixel size with growth temperature and duration, demonstrating that pixel size increases with both parameters.


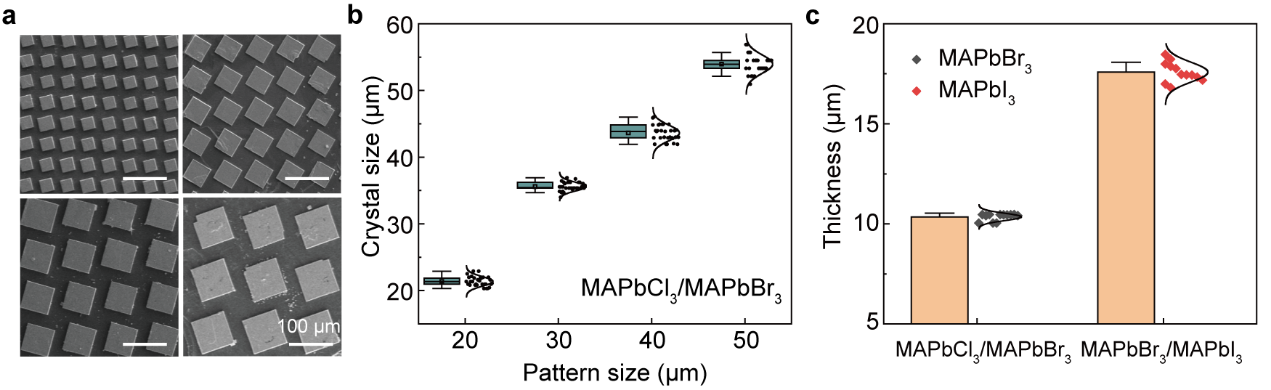


**Fig.** **S6** General selective epitaxial growth of MAPbCl_3_/MAPbBr_3_ perovskite single-crystal heterojunction arrays with diverse pixel dimensions. **a** SEM images of selective epitaxial MAPbBr_3_ single-crystal arrays with varying sizes. **b** Pixel size versus template opening size for MAPbBr_3_ single-crystal arrays epitaxially grown on MAPbCl_3_ substrates. **c** Statistical distribution of the vertical thickness for the epitaxially grown MAPbBr_3_ and MAPbI_3_ single crystals in the MAPbCl_3_/MAPbBr_3_ and MAPbBr_3_/MAPbI_3_ single-crystal heterojunction arrays with the pattern size of 40 μm, respectively


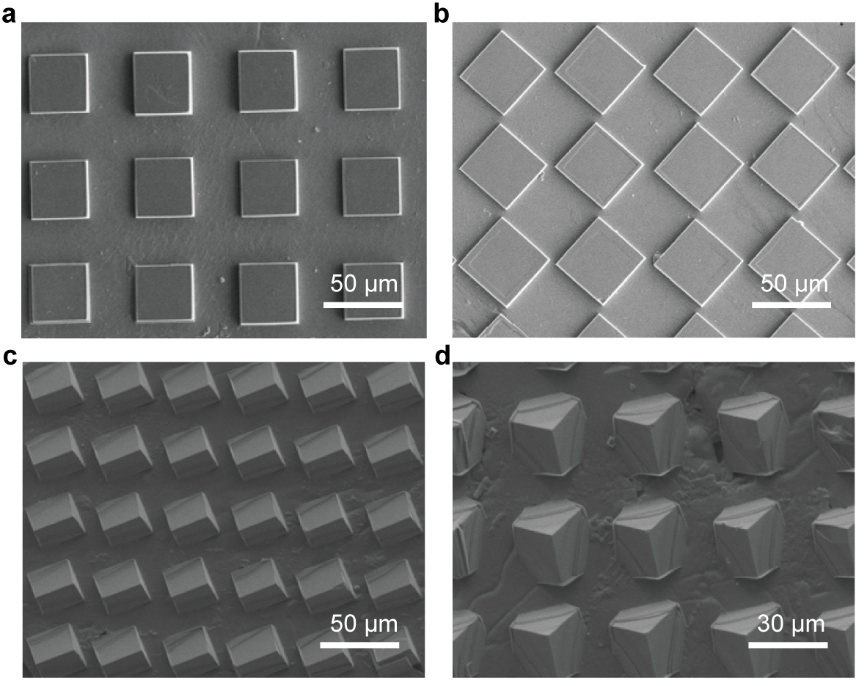


**Fig. S7** General selective epitaxial growth of MAPbCl_3_/MAPbBr_3_ perovskite single-crystal heterojunction arrays with diverse arrangement angles, and crystal orientation. **a, b** SEM images of epitaxial MAPbBr_3_ perovskite single-crystal arrays with different single-crystal orientations: **a** 0° and **b** 45°. **c, d** SEM images of epitaxial MAPbBr_3_ perovskite single-crystal arrays with different crystal orientations: **c** (110) and **d** (111). These results, achieved through a selective epitaxial growth method, demonstrate precise control over the size, angular alignment, and crystallographic orientation of the MAPbBr_3_ arrays on the MAPbCl_3_ substrate.


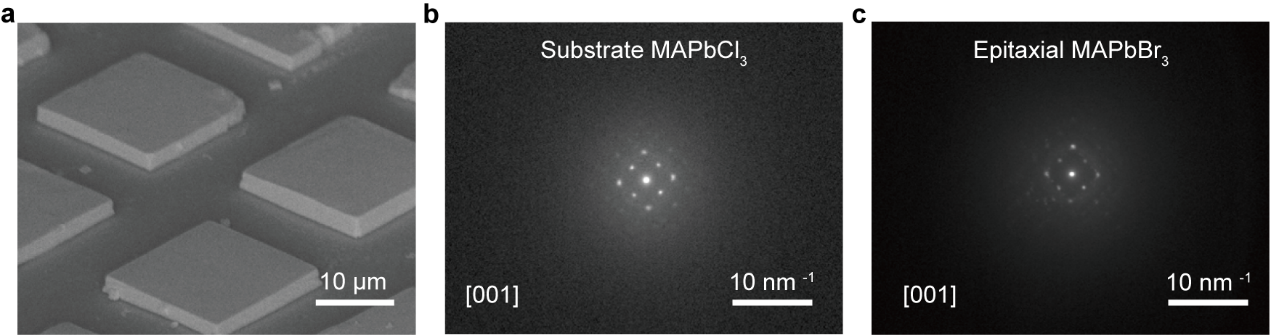


**Fig. S8** Structural and morphological characterization of low-temperature epitaxial perovskite arrays. **a** SEM image of a sheet-like MAPbBr_3_ array epitaxially grown on the MAPbCl_3_ substrate at 40 °C, revealing a morphological transition from pyramidal to cubic shapes. **b** TEM image of the MAPbCl_3_ substrate. **c** TEM image of the epitaxial MAPbBr_3_ array. The observed consistent crystal orientation validates that the heteroepitaxial relationship is maintained across the entire array, despite the evident evolution in crystal habit.


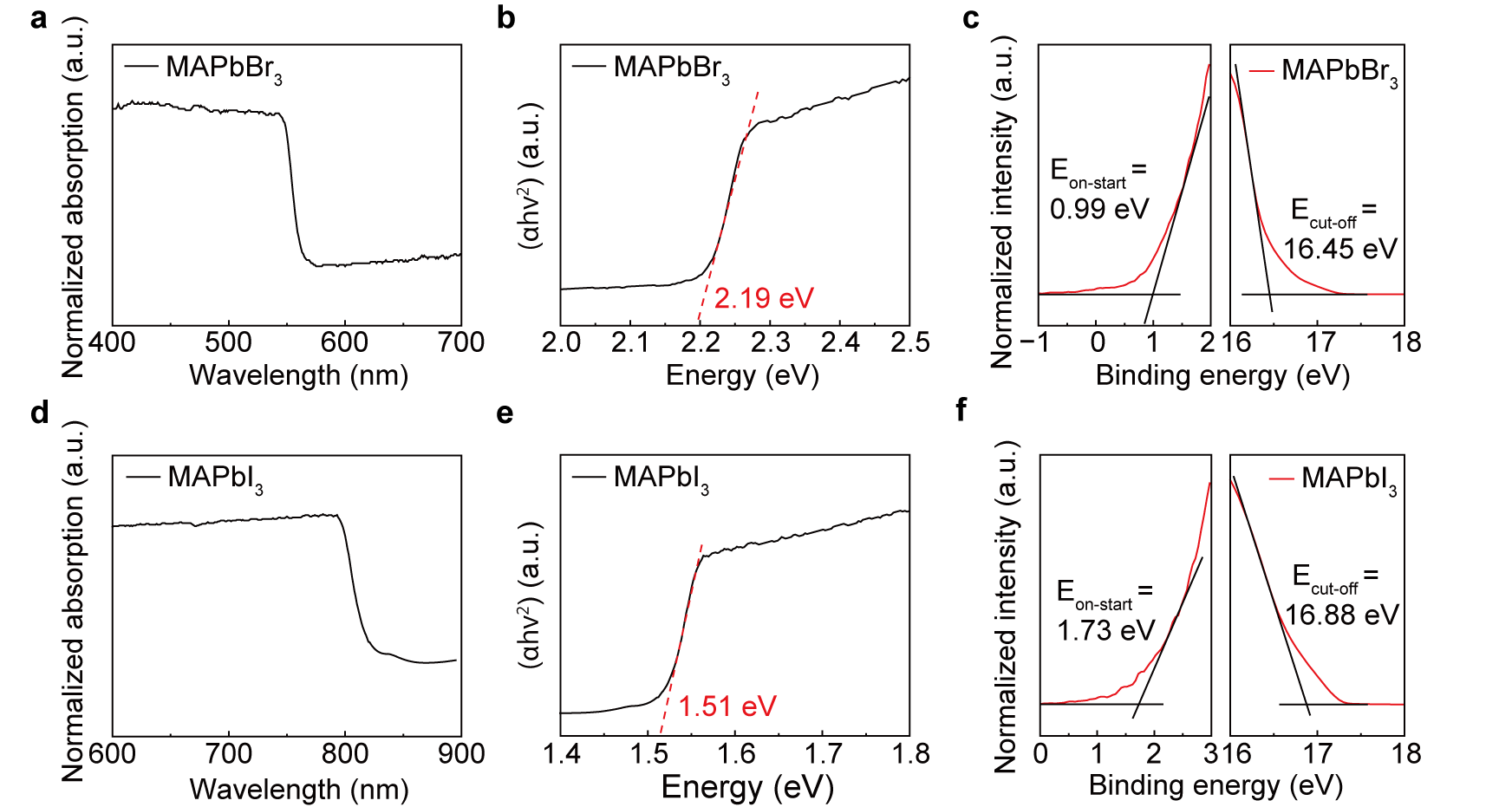


**Fig. S9** Absorption spectra and UPS of MAPbBr_3_ (**a, b, c**) and MAPbI_3_ (**d, e, f**) regions of the heterojunction crystal.


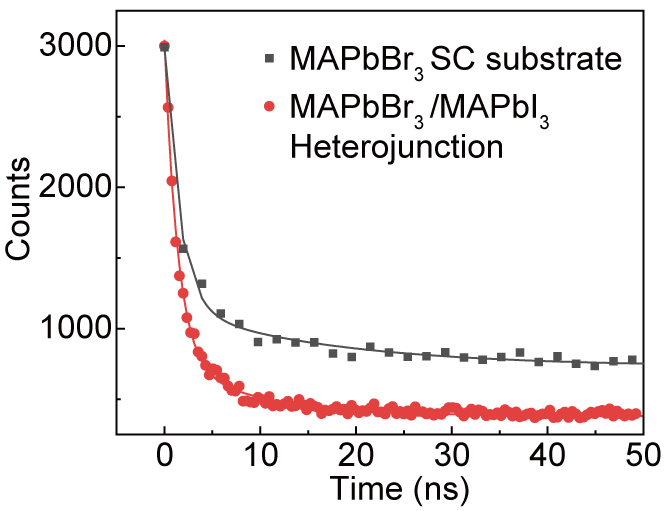


**Fig. S10** TRPL spectra of the heterojunction crystal at the transition regions. The decay curve was fitted with a biexponential function, yielding a fast decay time (τ₁) of 1.38 ns, a slow decay time (τ₂) of 9.28 ns, and an average carrier lifetime (τₐᵥ_g_) of 5.6 ns. In contrast, the MAPbBr_3_ single crystal exhibited τ₁ = 1.56 ns, τ₂ = 16.74 ns, and τₐᵥ_g_ = 11.52 ns. The substantial reduction in carrier lifetime for the heterojunction indicates the presence of an additional non‑radiative pathway for carrier extraction, which is attributed to the built‑in electric field at the heterojunction interface that efficiently separates photogenerated electrons and holes and suppresses radiative recombination.


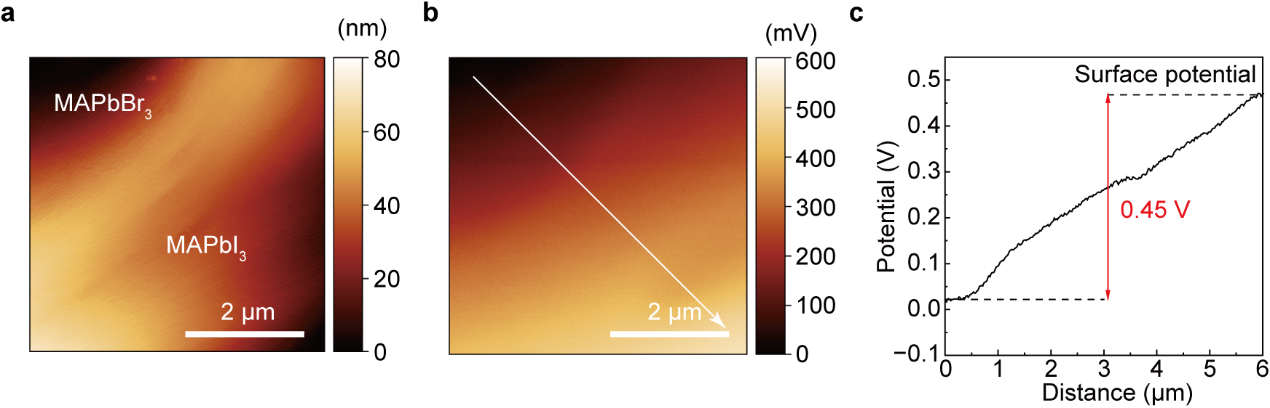


**Fig. S11 a** AFM measurement results on the surface of the heterojunction. **b** KPFM image of the MAPbBr_3_/MAPbI_3_ perovskite single-crystal heterojunction. **c** Surface potential of the MAPbBr_3_/MAPbI_3_ perovskite single-crystal heterojunction. A distinct surface potential difference of approximately 0.45 V was observed across the MAPbBr_3_/MAPbI_3_ heterojunction interface.


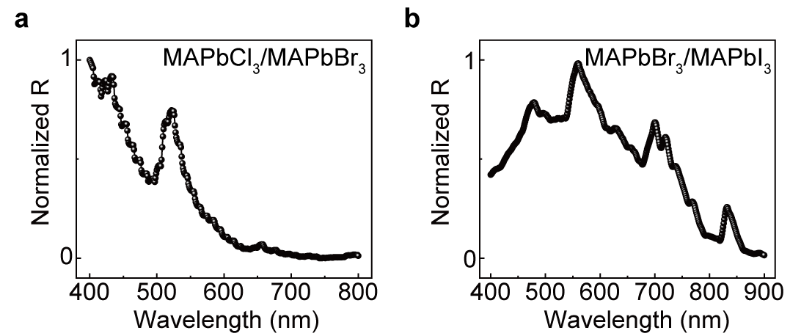


**Fig. S12** Full-spectrum response curve (bandwidth: 10 nm) of epitaxial single-crystal perovskite photodetector. **a** Normalized spectral responsivity curve of MAPbCl_3_/MAPbBr_3_ single-crystal heterojunction photodetector array. **b** Normalized spectral responsivity curve of MAPbBr_3_/MAPbI_3_ single-crystal heterojunction photodetector array.


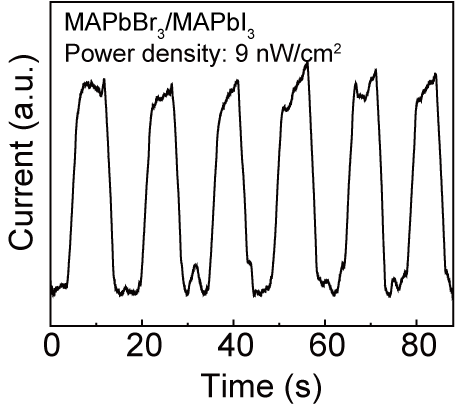


**Fig. S13** *I-t* curves of the MAPbBr_3_/MAPbI_3_ heterojunction photodetector under pulsed 532 nm illumination with the minimum light intensity of 9 nW/cm^2^.


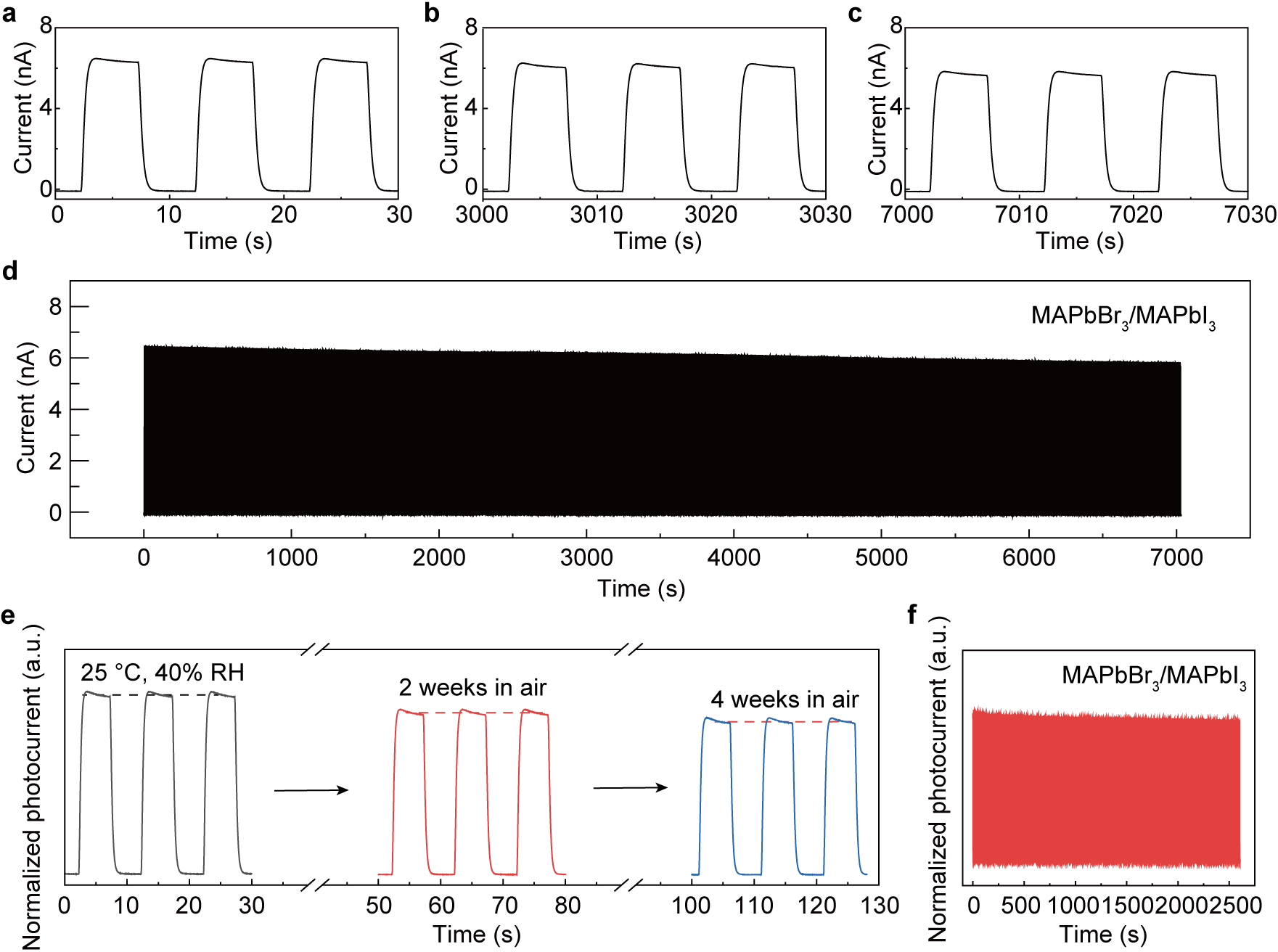


**Fig. S14** The photoresponse stability measured on the MAPbBr_3_/MAPbI_3_ single-crystal heterojunction photodetector under 532 nm illumination. **a** The initial *I-t* response of the device. **b** *I-t* response after 4% degradation in photocurrent from the initial value. **c** *I-t* response after 10% degradation in photocurrent from the initial value. **d** Long-term *I-t* response measured over 7000 seconds*.* **e** Comparison of the optoelectronic response changes after the device was stored in ambient air for four weeks. **f** *I-t* response measured after the device was stored in ambient air for four weeks.


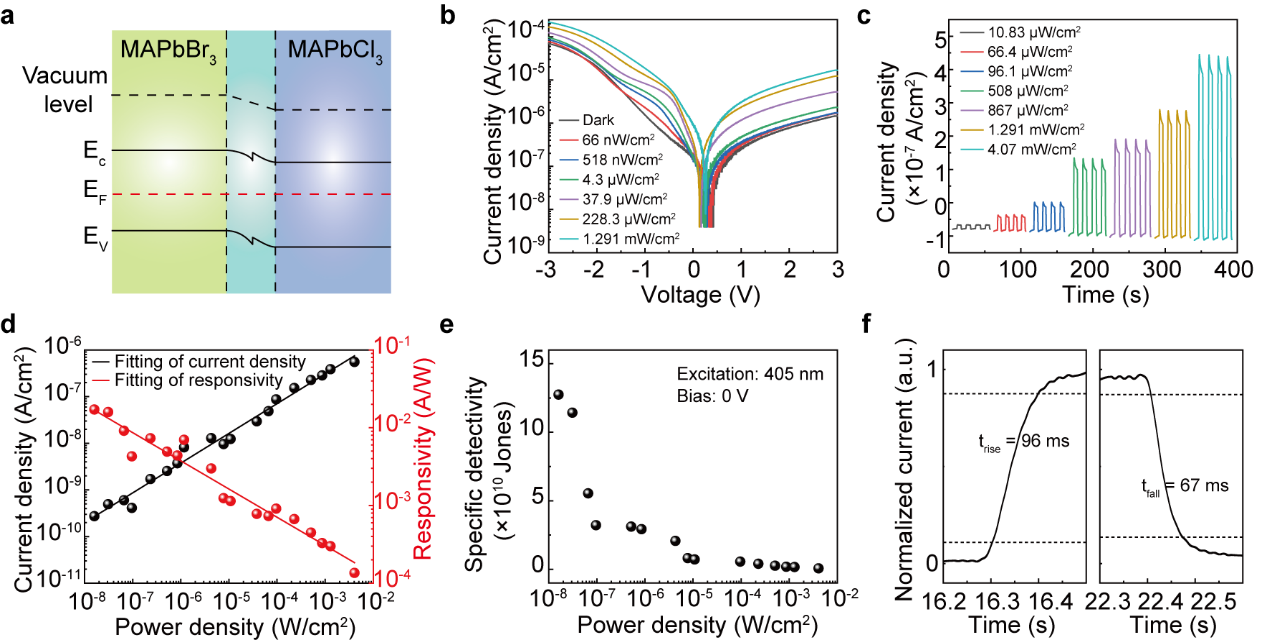


**Fig. S15** Optoelectronic response of self-powered photodetector based on the MAPbCl_3_/MAPbBr_3_ perovskite single-crystal heterojunction array. **a** Energy-band diagram of the heterojunction device, illustrating a type-II band alignment. **b** Current density-voltage (*J-V*) characteristics under varying light intensities. **c** Current density of the device under different light irradiation. **d** Photocurrent density and responsivity versus light intensity. The photocurrent shows a linear dependence on the light intensity and the responsivity is higher at lower light intensities, exhibiting a peak responsivity of 1.68 mA/W. **e** Specific detectivity (*D**) versus light intensity. The specific *D** reaches 1.27 × 10^11^ Jones under 1.6 × 10^-8^ W/cm^2^ weak light condition. **f** Response time of the heterojunction device under pulsed illumination. The self-powered photodetector array exhibits a fast response, with a rise time (*tᵣᵢₛₑ*) of 96 ms and a fall time (*t_fall_*) of 67 ms.


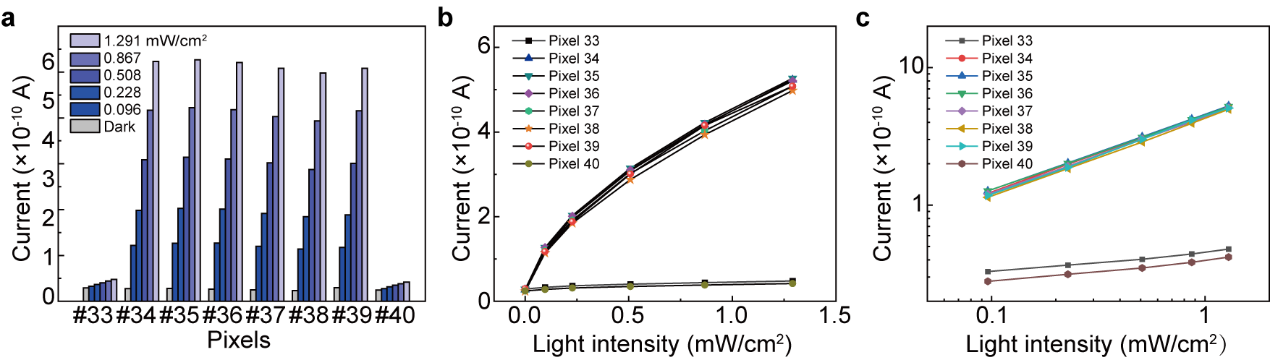


**Fig. S16** The statistical results of photocurrent as a function of light intensity for individual pixels within a specific row of the MAPbBr_3_/MAPbI_3_ perovskite single-crystal heterojunction photodetector array when the light source illuminates the device through a mask. **a** Current statistics under a range of incident light intensities for pixels 33-40. **b** Current versus light intensity for pixels 33-40. **c** Log-scale current as a function of light intensity for pixels 33-40.

**
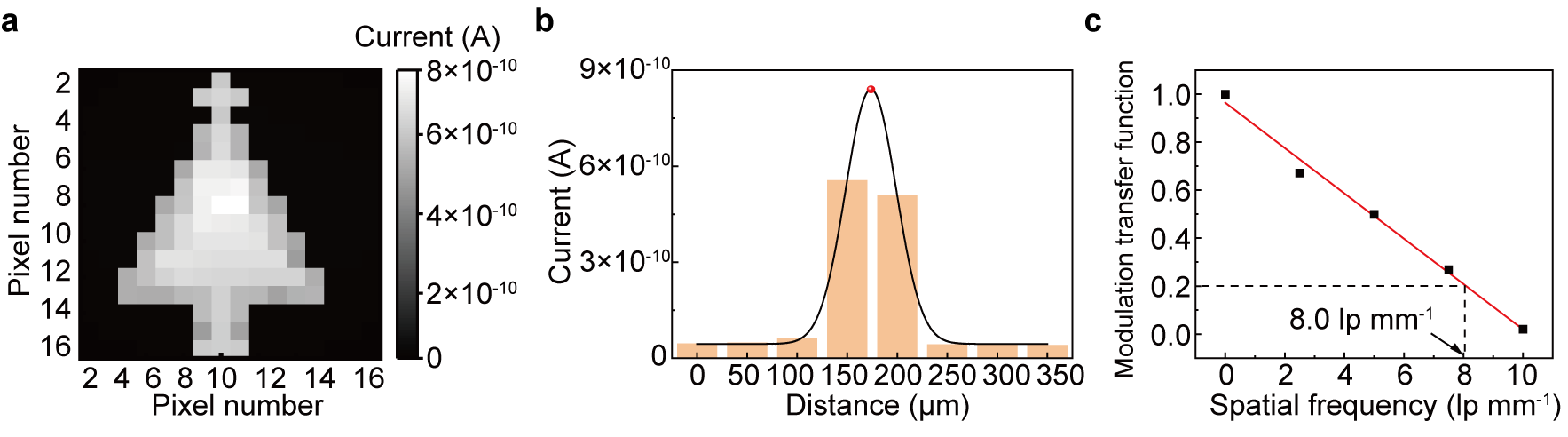
**

**Fig. S17** Imaging tests and performance characterization under complex scenarios. **a** Photocurrent mapping of the 16×16 image with a complex “Christmas tree” pattern. **b** Current distribution at different locations on the sample surface. **c** MTF of the device.

**Table S1** Performance comparisons of self-powered photodetectors based on perovskite single-crystal heterojunctions

**Supplementary References**

1. C. Liu, R. Wang, J. Liu, W. Xu, H. Zhang et al., High-performance self-driven MAPbI_3_^-^MAPbBr_3_ perovskite single crystal heterojunction photodetectors for ultra-sensitive weak light imaging. Adv. Opt. Mater. **13**(11), 2403123 (2025). <https://doi.org/10.1002/adom.202403123>
2. Y. Pan, X. Wang, Y. Liao, Y. Xu, Y. Li et al., Epitaxial perovskite single-crystalline heterojunctions for filter-free ultra-narrowband detection with tunable spectral responses. ACS Appl. Mater. Interfaces **14**(44), 50331–50342 (2022). <https://doi.org/10.1021/acsami.2c13126>
3. X. Zhang, L. Li, C. Ji, X. Liu, Q. Li et al., Rational design of high-quality 2D/3D perovskite heterostructure crystals for record-performance polarization-sensitive photodetection. Natl. Sci. Rev. **8**(10), nwab044 (2021). <https://doi.org/10.1093/nsr/nwab044>
4. L. Xue, X. Wang, Y.-Z. Pan, M. Luo, Y. Xu et al., Fast response, high spectral rejection ratio, self-filtered ultranarrowband photodetectors based on perovskite single-crystal heterojunctions. ACS Appl. Mater. Interfaces **15**(46), 54050–54059 (2023). <https://doi.org/10.1021/acsami.3c10559>
5. C. Liu, H. Chen, P. Lin, H. Hu, Q. Meng et al., Optimized photoelectric characteristics of MAPbCl_3_ and MAPbBr_3_ composite perovskite single crystal heterojunction photodetector. J. Phys. Condens. Matter **34**(40), 405703 (2022). <https://doi.org/10.1088/1361-648X/ac84bc>
6. Y. Guan, C. Zhang, Z. Liu, Y. Zhao, A. Ren et al., Single-crystalline perovskite p–n junction nanowire arrays for ultrasensitive photodetection. Adv. Mater. **34**(35), 2203201 (2022). <https://doi.org/10.1002/adma.202203201>
7. F. Cao, Z. Li, X. Liu, Z. Shi, X. Fang, Air induced formation of Cs_3_Bi_2_Br_9_/Cs_3_BiBr_6_ bulk heterojunction and its dual-band photodetection abilities for light communication. Adv. Funct. Mater. **32**(46), 2206151 (2022). <https://doi.org/10.1002/adfm.202206151>
8. Y.-W. Hsiao, B.S. Cheng, H.-C. Hsu, S.-H. Wu, H.-T. Wu et al., Vertical-type 3D/quasi-2D n-p heterojunction perovskite photodetector. Adv. Funct. Mater. **33**(21), 2300169 (2023). <https://doi.org/10.1002/adfm.202300169>
